# High-latitude platform carbonate deposition constitutes a climate conundrum at the terminal Mesoproterozoic

**DOI:** 10.1038/s41467-024-46390-w

**Published:** 2024-03-06

**Authors:** Michiel O. de Kock, Ingrit Malatji, Herve Wabo, Joydip Mukhopadhyay, Amlan Banerjee, L. P. Maré

**Affiliations:** 1 Department of Geology, University of Johannesburg, PO Box 524, Auckland Park, South Africa; 2 Department of Earth and Environmental Sciences, Indian Institute of Science Education and Research, Odisha, Berhampur India; 3 Geological Studies Unit, Indian Statistical Institute, 203 B. T. Road, Kolkata, West Bengal India; 4 Council for Geoscience, Private Bag X112, Pretoria, South Africa

**Keywords:** Palaeomagnetism, Palaeoclimate, Precambrian geology, Stratigraphy

## Abstract

During the Mesoproterozoic Era, 1600 to 1000 million years ago, global climate was warm with very little evidence of glaciation. Substantial greenhouse warming would have been required to sustain this ice-free state given 5-18% lower solar luminosity. Paleomagnetic data reported here place voluminous ca. 1.2 Ga shallow marine carbonate deposits from India at an unexpectedly high latitude of around 70° from the equator. Previous studies noted high latitudes, but their implication was never considered. Here, we evaluate the temporal-latitudinal distribution of neritic carbonate deposits across the Proterozoic and identify similar deposits from North China that together with those from India are seemingly unique to the late Mesoproterozoic. A uniformitarian interpretation implies that this is cold-water carbonate deposition, but facies similarity with low-latitude neritic deposits rather suggests a hotter climate and elevated polar ocean temperatures of 15–20° or higher. This interpretation represents a climate conundrum that would require much greater greenhouse warming than documented for the Mesoproterozoic.

## Abstract (web summary)

Deposition of 1.2-billion-year-old Indian limestone in shallow seas near the poles imply balmy conditions of more than 15 °C and significantly higher atmospheric greenhouse gas concentrations, which expands the spectrum of Earth’s climatic extremes.

## Introduction

**Fig. 1:**
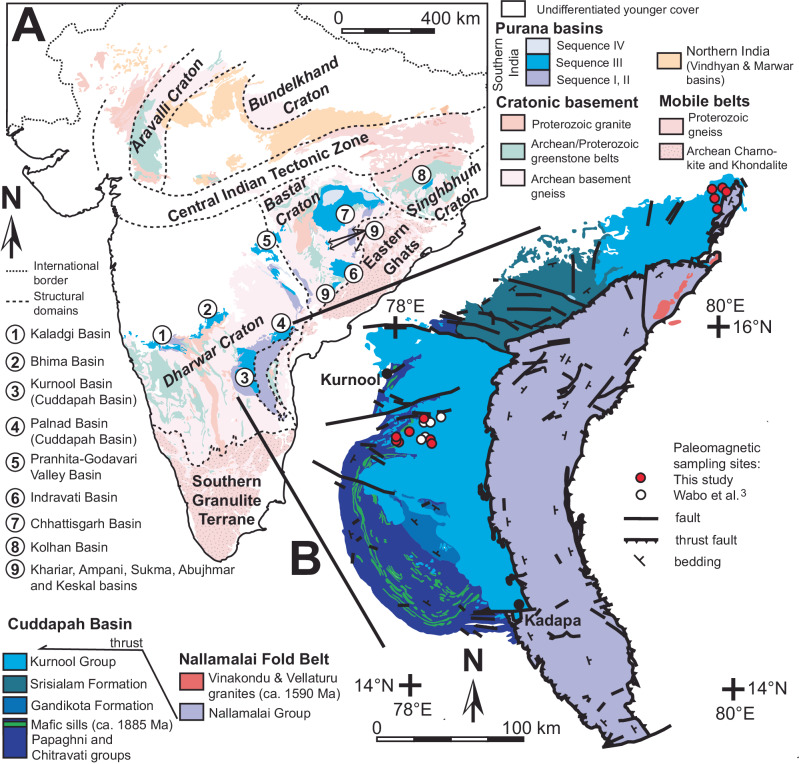
Geology of India and the Cuddapah Basin. **A** Simplified Precambrian geology and structural domains of India with the locality of the Purana basins shown. **B** Geology of the Cuddapah Basin with paleomagnetic sampling localities. Modified from refes.^1^ and Dasgupta et al.^57^.

**Fig. 2:**
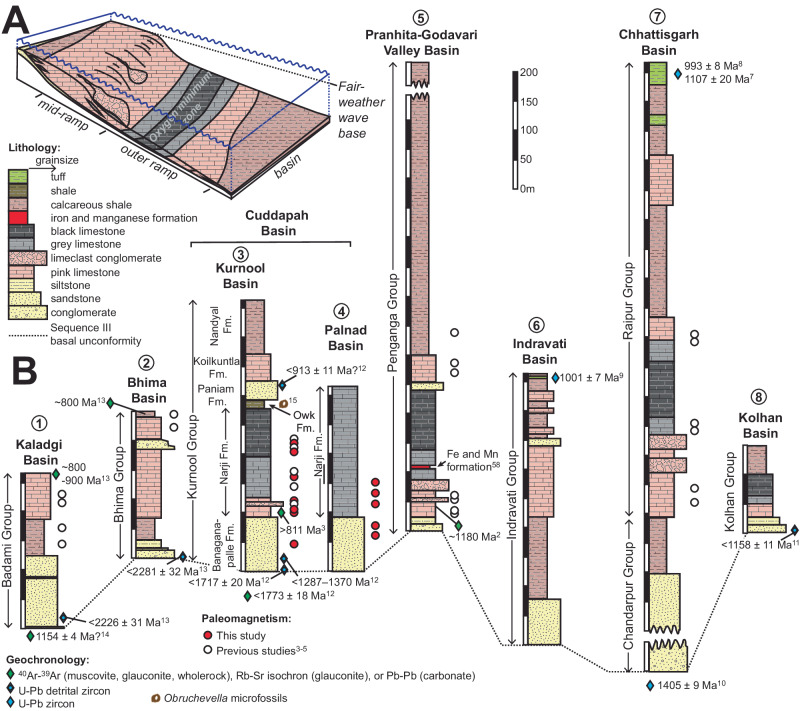
The Purana platform of southern India. **A** Paleoenvironmental reconstruction of the Purana platform based on Mukhopadhyay et al.^11^ and Gutzmer and Beukes^58^. **B** Correlation of Sequence III across southern India with age constraints and paleomagnetic sampling positions (Modified from Wabo et al.^3^). Stratigraphic profiles are numbered by location as per Fig. 1. These carbonates were mostly deposited below wave base and are bedded lime mudstones with spatially limited columnar and branching stromatolite occurrences, calcareous shales, and locally developed lime-clast debris-flow conglomerates and calciturbidites in distally steepened settings. Lime-mudstones are overlain by calcareous shale and in some areas by another cycle of carbonate deposition. Limestone exhibits stratigraphically controlled cycles of colour variation from brown-pink to black through steel gray and again to brown through steel gray in some of the basins, while only steel gray limestone is preserved in others. The Pranhita-Godavari Valley Basin preserves a unique distal transect of the platform. Here a two metre-thick banded iron-manganese formation is developed in siliceous gray limestone due to upwelling circulation at the platform edge^58^.

Proterozoic basins from cratonic India are collectively referred to as the ‘Purana’ basins^1^ and host several unconformity-bound, relatively undeformed and unmetamorphosed sedimentary sequences (Fig. 1a). One such unconformity bound sequence (i.e., Sequence III) is preserved in most of the Purana basins of southern India and consists of shallow-marine sandstone followed by a carbonate ramp succession of limestone and calcareous shale^2,3^ (Fig. 2). Several terms exist for neritic carbonate deposits based on geometry (i.e., ramps, rimmed shelfs, and open shelfs), but we generically use the term “platform”. Past paleomagnetic studies support the correlation of the Purana platform succession across southern India and indicate that it was deposited at high-latitude^3–5^, but contradictory results are reported by Goutham et al.^6^ .Together with paleomagnetic data, we discuss the high-latitude, characteristics, and the climatic implications of the Purana platform within the context of known paleolatitude constraints for shallow-marine carbonate deposition since the Proterozoic.

The minimum age limit of the Purana platform is directly constrained by U-Pb zircon crystallization ages from tuffaceous units in the Chhattisgarh and Indravati basins to predate 993 ± 8 million years (Ma) and 1001 ± 7 Ma, respectively^7–9^, and to postdate 1405 ± 9 Ma in the Chhattisgarh Basin^10^ (Fig. 2b). An ^40^Ar/^39^Ar glauconite age of ca. 1180 Ma provides a minimum constraint for the Purana platform in the Pranhita-Godavari Basin^2^. Detrital zircon ages of 1158 ± 11 Ma from the Kolhan Group (on the Singhbhum Craton) suggest that it, too, is likely coeval with similar facies in the Pranhita–Godavari Basin^11^ (Fig. 2b). The timing of the platform’s deposition is even less certain in the Kaladgi, Bhima and Cuddapah basins, but is broadly assigned to the latest Mesoproterozoic to early Neoproterozoic by means of U-Pb ages from detrital zircon grains^12,13^, ^40^Ar/^39^Ar whole rock ages^14^, ^40^Ar/^39^Ar ages and Rb-Sr isochron ages on glauconite^3,13^, Pb-Pb ages on carbonate^13^, and the presence of microfossils^15^ (Fig. 2b).

Within the Cuddapah Basin, the platform is represented by the Kurnool Group, which is preserved in two erosional remnants: the Kurnool and Palnad basins (Fig. 1b). Preservation is more complete in the Kurnool Basin, where the group comprises the Banaganapalle Quartzite, Narji Limestone, Owk Shale, Paniam Quartzite, Koikuntla Limestone and Nandyal Shale from the base upwards (Fig. 2b). The Narji Limestone is mostly micritic with laterally persistent tabular beds of gray limestone with intercalated siliciclastic units and pink limestone near its base. It grades into thinly bedded black limestone and eventually into ochre-coloured shale of the Owk Formation^16^. Helically coiled *Obruchevella* microfossils from the Owk Formation suggest a Neoproterozoic to Early Cambrian age^15^ (Fig. 2b). The Paniam Formation overlying the Narji Limestone yielded a single 913 ± 11 Ma zircon grain^12^, but the next youngest age population from several grains at 1717 ± 20 Ma is a more conservative maximum depositional age estimate. Paleomagnetic data from the Narji Formation suggest an age of ~1.2 Ga^3^.

Carbonate accumulation in the ocean today extends as a continuum across low to high latitudes and from shallow marine to pelagic environments. Today, low-latitude warm-water platforms are distinct from high-latitude temperate-to-cold water platforms in terms of biotic constituents^17,18^. Low-latitude platforms are characterized by a photozoan association of autotrophic carbonate producers such as green calcareous algae, invertebrates with hermatypic coral and precipitates of lime-mud and ooids. Modern cold-water platforms are largely comprised of heterozoan bioclasts with ahermatypic coral, coralline algae and benthic invertebrates, while precipitates like carbonate mud and oolitic grains are poorly documented^18^. Carbonate mud, which is usually a minor component of high-latitude Phanerozoic carbonate deposits^17^, are interpreted as the product of skeletal fragment breakdown^19^. Volumetrically, warm-water neritic environments within ~30° of the equator are the principal settings for carbonate production and preservation during the Phanerozoic^20^. The divide between modern cold- and warm-water platforms is around seawater temperatures of 20 °C^21^.

Precambrian platforms are generally considered analogous to modern warm-water facies^22^ and preferential tropical deposition is assumed by palaeogeographers^23^. This assumption may be false. Precambrian platforms are devoid of bioskeletal grains, coral and bryozoan bioherms, and are instead dominated by stromatolites and participates like lime-mud with a subordinate proportion of ooid grains, but also aragonite fans in the Precambrian, and molar-tooth structures in the later part of the Proterozoic^22^. Cold-water Precambrian platforms are not well-described and could be difficult to recognize given the modern Metazoan-based definition. They should, however, share those characteristics of modern cold-water facies that are not Metazoan-based. Precambrian high-latitude cold-water carbonate facies are thus likely lower volume deposits compared to tropical counterparts deposited at the same time. In addition, significant reworking (i.e., clastic textures) and minor carbonate mud components are expected, and they are likely to contain interbedded glacial deposits, ice-rafted debris, local hardgrounds, and glendonites^17^.

Here, we report carbonate deposition at high latitude with paleomagnetic data for the Kurnool Group based on more extensive sampling compared to previous studies (Supplementary Fig. 1). We collected oriented limestone, sandstone, and shale core plugs by standard methods from the Banaganapalle and Narji formations for paleomagnetic and the first rock magnetic analyses for these rocks to better constrain their magnetization and paleolatitudinal setting (see Methods). We compare this with published paleomagnetic results from southern India and constrain carbonate deposition at high latitude around ~1.2 Ga. We further compile paleolatitudes for Proterozoic carbonate platforms from paleomagnetic results listed in the Global Paleomagnetic Database^24^ and those published recently but not yet incorporated into this database to evaluate the temporal and spatial context of these southern Indian carbonate successions (see Methods). High-latitude (i.e., >50° latitude) neritic carbonate deposition is seemingly unique to the late Mesoproterozoic based on this compilation. Given the facies characteristics, we interpret the high-latitude carbonate deposition as a proxy for polar ocean water temperatures of at least above 15 °C (but likely higher than 20 °C) and for greenhouse climatic conditions that required higher pCO_2_ (and other greenhouse gasses) than previously considered for the Mesoproterozoic.

## Results

Here we provide a summary of the demagnetization and rock magnetic results. For a more detailed account, see Supplementary Notes.

**Fig. 3:**
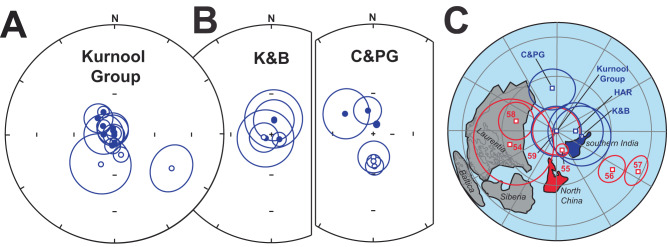
Summary of paleomagnetic results. **A** Steep dual polarity remanence directions were obtained by demagnetization of samples from the Kurnool Group (This study). All directions are shown in tilt-corrected coordinates. Symbols: closed = down, open = up. Ellipses = α_95_ confidence cones about site means. **B** Steep dual polarity remanence directions were previously obtained by demagnetization of samples from Kaladgi and Bhima basins (K&B) ref. ^5^, Chhattisgarh and Pranhita-Godavari Valley basins (C&PG) ref. ^4^. All directions are shown in tilt-corrected coordinates. Symbols: closed = down, open = up. Ellipses = α_95_ confidence cones about site means. **C** Reconstruction of southern India and North China based on poles from late Mesoproterozoic high-latitude platform carbonate units relative to the well-established core of Rodinia (i.e., Laurentia, Siberia, and Baltica). Depicted poles are listed in Supplementary Notes. Southern India was reconstructed by a rotation of 64° around an Euler pole located at 0°N and 337°E. North China is rotated by 47° around an Euler pole located at 0°N and 60°E.

A small number of samples (mostly from the Palnad Basin) did not respond well to demagnetization and revealed incoherent remanence directions that are excluded from further discussion. With demagnetization below 350 °C, our samples are characterized by shallow north-down components (Supplementary Table 1, Supplementary Figs. 2–4). Similarity of these relatively low temperature shallow north-down components with the remanence reported by Goutham et al.^6^ is noted (Supplementary Fig. 6) and is suspected to represent a record of the modern geomagnetic field. The majority of samples, however, yield steep components during thermal demagnetization to either 420 °C or 660 °C. These were southeast-up, or northwest-down (Supplementary Table 1, Supplementary Figs. 2–4). Thermomagnetic curves confirm haematite as magnetic carrier of the high-temperature components in pink limestone and magnetite in black and gray limestone (Supplementary Figs. 5 and 7). The identified components are similar to and combined with those from a previous study of the Narji Limestone^3^ (Fig. 3a). Together the data yield a paleopole at 26.4°N and 66.6°E with A_95_ = 13.3° (Fig. 3c). Formal evaluation of the reversal test is inconclusive due to the small number of sites that record the southeast-up component. However, a positive fold test illustrated that these steep directions predate ~1.0 Ga deformation^3^ (Supplementary Notes). In addition, an intraformational conglomerate test imply that the magnetization is primary^3^ (Supplementary Notes). Our pole is different from the Phanerozoic apparent polar wander path of Gondwana (Supplementary Fig. 8a) and differs from Precambrian key paleomagnetic poles from India at 1465 Ma, 1075 Ma and 770 Ma, which all place India at lower latitudes (Supplementary Fig. 8b). It is, however, similar to the 1192 ± 10 Ma Harohalli alkaline dykes pole^25^ (HAR in Fig. 3c). The implication is that the age of magnetization and the Purana platform is likely removed from the current maximum (1405 ± 9 Ma) and minimum (1001 ± 7 Ma) age constraints, but closer to 1.2 Ga as also indicated by the ~1180 Ma glauconite age from the Pranhita–Godavari Valley. We tentatively assign the 1192 ± 10 Ma age to our pole but remain cognizant of the wider possible age range.

## Discussion

Limestone of the Purana platform from the Chhattisgarh and Pranhita–Godavari Valley basins and those from the Kaladgi and Bhima basins are also characterized by steep north-down and south-up remanence directions that are stable either above 600 °C or up to 420–460 °C^3,5^ (Fig. 3b). Our Kurnool Group pole is similar to poles from these previous studies (K&B and C&PG in Fig. 3c). The pole from the Chhattisgarh and Pranhita–Godavari Valley basins is considered primary based on a record of reversals, a positive regional fold test^4^, and most importantly, a positive intraformational conglomerate test^4^. The Kaladgi and Bhima basins also record a reversal, but its age of magnetization is not constrained by field tests^5^. Our data indicate a paleolatitude of 72° ± 12° for southern India during Kurnool Group deposition (Fig. 3c), which is comparable to the 72° ± 13° and 83° ± 16° obtained from carbonate facies of the Chhattisgarh and Pranhita–Godavari Valley basins and the Kaladgi and Bhima basins^4,5^. The Purana platform thus developed at least partly at high latitude over much of Peninsular India.

**Fig. 4:**
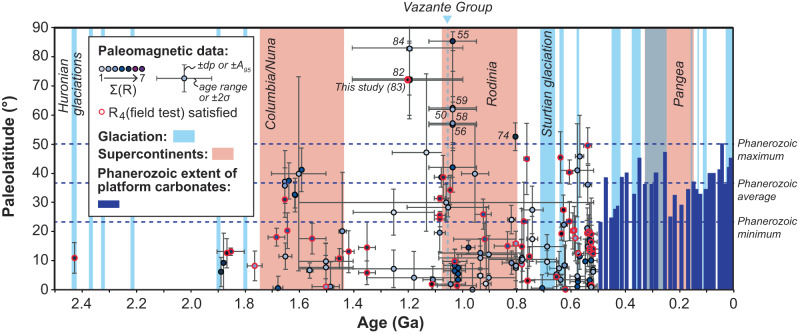
Paleolatitude of Proterozoic platform carbonates. Proterozoic paleolatitudes are compared to the maximum latitudinal extent of platform carbonates during the Phanerozoic (after Kiessling et al.^20^). Nine studies (numbered to correspond to numbering in Supplementary Table 3) display paleolatitudes >50° during a time interval that is largely free of glaciation apart from a possible occurrence in the Vazante Group of the São Fransisco craton. Proterozoic studies are rated for their quality (R) after ref. ^56^, and those whose age of magnetization is supported by a field test are outlined in red. Horizontal error bars represent platform age ranges or 2σ where absolute ages are available. Vertical error bars are either represented by ±dp, the minimal axis of the oval of 95% confidence for the virtual geomagnetic pole, or ±A_95_, the radius of the circle of 95% confidence for the paleomagnetic pole.

Our compilation of paleolatitudes (Supplementary Table 3) reveals only a few studies that fall outside the latitudinal extent of their Phanerozoic counterparts^20^ (Fig. 4). Nine studies show paleolatitudes greater than 50° and occur either at ~1200–1000 Ma or ~800 Ma. The older grouping is represented by the Purana platform (as discussed above), but also by the Nanfen, Xinxing and Liulaobei formations of the North China craton^26–28^. In addition, the Jingeryu Formation was recently interpreted as correlative strata of the Nanfen and Xinxing carbonate deposition in the polar region at 1.1 Ga^29^. Paleolatitudes from the Purana platform and the lower Nanfen Formation and its equivalents are between 59° and 90°. The upper parts of the lower Nanfen Formation and the middle member of the Nanfen Formation yield lower, albeit still high paleolatitudes of 49°–64°. Paleolatitudes between 39° and 85° have previously been reported from the Jingeryu Formation^26^. The paleomagnetic constraints from the Purana platform are supported by field tests and dual-polarity directions are recorded. The lower part of the Nanfen Formation and its equivalents also record magnetic reversals. The identification of extreme high-latitude carbonate deposition within a broadly similar timeframe from two cratonic blocks with a comparable facies change from purplish-pink to gray limestone is suggestive of a shared history, and we reconstruct India and North China as near neighbours during the assembly of Rodinia (Fig. 3c). Such a near-neighbour relationship has been noted before, albeit in a different configutaion^30^.

The ~800 Ma occurrence of high-latitude carbonates are represented by a unit from South China: the Madiyi Formation^31^ at 52° ± 5°. The study is unsupported by field tests, but does record reversals. It further overlaps within uncertainty with the latitudinal extent of Phanerozoic carbonates, thus representing a less convincing argument for extreme high-latitude deposition.

Most Proterozoic platforms conform to modern deposition at <50° latitude (Fig. 4). The absence of platforms at high latitude for much of the Proterozoic, could be the product of a preservation age bias. The likelihood that primary magnetizations are recorded also decreases with age, even if deposits are preserved. As such, our compilation reflects a paucity of data before 1.7 Ga (Fig. 4). The deposition and ultimate preservation of carbonate platforms could also be affected by the supercontinent cycle^32^. Other biasing factors are the presence of continents near the poles and the area on the globe at high latitude compared to low latitude. If high-latitude neritic carbonates were more commonplace, then they were not preserved. At face value, however, extreme high-latitude platforms are limited to ~1200–1000 Ma. They are not considered cold-water platforms, as the facies document a laterally extensive and distally steepened ramp that is rich in carbonate mud^33^, devoid of interbedded glacigene deposits and without documented glendonites. The facies are more typical of warm-temperate to warm conditions, and thus likely a proxy for hotter global climatic conditions. We note that an extraordinary climate condition was also recently suggested for the 1.1 Ga high-latitude carbonate platform(s) of North China^29^.

Alternatively, the development of non-cold-water but high-latitude platform carbonates require a non-actualistic explanation. A high-obliquity Earth^34^ could possibly explain the occurrence, but would also imply enhanced seasonality and trade winds near an arid equator. However, normal climatic zonation and low-obliquity is suggested by the distribution of large evaporite deposits during the Proterozoic^35^, albeit not unambiguously^34^. Steep magnetic inclinations can also be recorded at low- to mid-latitudes if the geomagnetic field had large non-dipole contributions or if the field was dominated by an equatorial dipole^36^. Such explanations are, however, unlikely given the evidence for a geocentric axial dipole during the late Mesoproterozoic^35^.

The Mesoproterozoic has long been considered a warm time based on the general absence of glaciation at a time of lower solar luminosity^37–39^. There are, however, some reports of possible glaciation at 1.0–1.1 Ga from the São Fransico craton^40^, but the occurrence is complicated in that the craton was located at low-paleolatitude during this time^23^. According to zero methane climate simulations for the Mesoproterozoic, pCO_2_ at 12 times PAL would have produced a glacial climate, while pCO_2_ at 24 times PAL would be needed to produce ice-free continents (but not at the poles)^41^. Current quantitative constraints on late Mesoproterozoic pCO_2_ are associated with large uncertainty. Estimates from paleosols^42,43^ put pCO_2_ at 10–20 times PAL, suggesting that warming possibly required other greenhouses gases or changes in Earth’s albedo. Although estimations from paleosols are disputed^44^, there is support from calcified cyanobacterial sheaths^45^. Paired carbon isotopic data suggest a possible range in pCO_2_ of 10–200 times PAL from ~1.4 Ga microfossils^46^, and 2 to 50 times PAL from ~1.6 Ga limestones^47^. At this stage it is unclear if elevated pCO_2_ alone provided the required warming to ensure largely ice-free conditions, but it is likely that other greenhouse gases also played a role^48^. Platform carbonate deposition at latitudes around 70° or higher that were rich in carbonate mud and without documented glendonites or interbedded glacigene deposits would, however, imply polar ocean temperatures higher than −1.5 °C and may have required more than 24 times PAL pCO_2_ or significantly higher levels of other greenhouse gasses. Polar ocean temperatures may have been higher than ~20 °C if our comparison with modern warm-water analogues is correct^21^. Such a greenhouse climate can perhaps be compared with the mid-late Cretaceous, one of the best-studied warm intervals of the Phanerozoic, which saw annual low-latitude sea surface temperatures of ~35 °C with a minor temperature gradient extending to polar waters at ~13–21 °C^49–51^, but platform carbonates here only reached latitudes as high as ~40° (Fig. 4).

In conclusion, paleomagnetic data reveal development of laterally extensive carbonate ramps at very high latitude during the late Mesoproterozoic across much of southern India and North China. There is no evidence that suggests that these are cold-water carbonate facies, and we interpret the facies as a proxy for greenhouse climatic conditions that required higher atmospheric pCO_2_ (and other greenhouse gasses) than previously considered for the Mesoproterozoic. Conditions had to allow for largely ice-free poles, but also for polar ocean temperatures possibly higher than 20 °C. Explaining Mesoproterozoic high-latitude carbonate facies, however, goes beyond ocean temperature alone. Controls like carbonate saturation, ocean circulation or continental configuration should be considered in the future as ancient continental reconstructions become more granular.

## Methods

### Sampling

All samples were collected with the necessary permissions in collaboration with staff of the Geological Studies Unit at the Indian Statistical Institute, Kolkata.

The Kurnool Group of the Cuddapah Basin was sampled for paleomagnetism, rock magnetism, and petrography. The Banaganapalle Formation and the Narji Formation was sampled from both the Kurnool as well as the Palnad basins (Supplementary Fig. 1). Core samples were collected using a portable petrol-powered drill and oriented using both magnetic and sun compasses. Structural orientation of beds was recorded. Small hand samples were also collected from all the localities that were drilled. At two localities it was not possible to drill because of concerns about antagonizing the local community, and oriented block samples that were later plugged using a bench top drill-press were collected instead. No additional hand samples were collected from these localities. One oriented core plug was drilled from each block sample. Oriented cores were cut into ~2 cm long cylindrical specimens with smooth and parallel top and basal surfaces. Selected hand samples, duplicate specimens and cut offs were used to prepare polished thin sections or were used for rock magnetic characterization.

In the Kurnool Basin (Supplementary Fig. 1a) site KBA was sampled from a roadcut in immature sandstone and conglomerate of the lower Banaganapalle Formation that dip shallowly to the south. Seven cores were collected here. Site KND, with 10 oriented cores represents pink calcareous shale from the transition between the Banaganapalle and Narji formations. Light purplish gray limestones were collected at sites KNA and KNB (11 and 8 cores, respectively), both in the lower stratigraphic levels of the Narji Formation. Sandstone lenses are interbedded with the limestone at site KNA, where bedding is essentially horizontal. Site KNB near the village of Ambapurum is stratigraphically somewhat below site KNA in light gray to pink ripple-marked limestone. Eight cores were collected from flat bedded light gray limestones at KNC. Twenty cores of planar laminated black limestones (i.e., KNE and KNF) were collected from different parts of a large quarry outside Rama Krishna Purum. In the Palnad Basin (Supplementary Fig. 1b) two sites were sampled within the Banaganapalle Formation (PBA and PBB), and three sites from the Narji Formation (PNA, PNB, and PNC). Site PBA represents fine-grained sandstone and siltstone with well-preserved ripple cross-lamination as well as wave ripple and interference ripple marks. Six oriented block samples were collected here. Five oriented core samples were collected from a course-grained sandstone at site PBB. A dark gray limestone (PNA, 6 cores) and a light gray thinly bedded limestone (PNC, 8 cores) display obvious signs of deformation with a well-developed lineation on bedding planes. There is also abundant stylolitization developed at both localities. At site PNB, a planar bedded dark gray limestone is not obviously affected by deformation, and 5 oriented block samples were collected.

### Paleomagnetism

Specimen were magnetically characterized through measurement of the Natural Remanent Magnetization (NRM) using the Superconducting Cryogenic rock magnetometer 2 G 760-4 K (SQUID) with an automatic sample changer at the University of Johannesburg paleomagnetic laboratory. The components of magnetization that makes up the NRM were identified and quantified through stepwise alternating field and thermal demagnetization. The alternating field (AF) demagnetization utilized coils that are in line with the superconducting rock magnetometer and were used to demagnetize low coercivity magnetic components that were present in the samples. The samples were exposed to AF field strengths of 2.5, 5.0, 7.5, and 10.0 mT. Thermal demagnetization was achieved by heating specimen from 100 °C2500–680 °C at decreasing intervals using a model TD-48SC shielded furnace. Demagnetization generally consisted of 15–20 steps. Remanence components were identified and quantified by means of principal component analyses^52^ using the software PMag 3.1.0^53^ and PmagPy^54^. Only components based on three or more demagnetization steps and with a maximum angular deviation (MAD) less than or equal to 15° were considered for further interpretation. Component means were used to calculate VGPs (Virtual geomagnetic poles) and these VGPs were grouped to calculate grand mean poles. All visualizations of calculated and published poles utilized GPlates 2.2^55^.

Selected Narji Formation sample material of this study (i.e., KNE01, PNA02, KND02) and a previous study^3^ (i.e., KPA07, KPB06) were crushed with an agate mortar and pestle, and 1 g fractions of each sample consisting of a third of the material being >1 mm and the remainder being <0.71 mm were prepared. The changes in magnetic susceptibility (κ) with changes in temperature (T) of these samples were determined in air from 30 ° to 700 °C at a standard heating rate of ~10 °C per minute using a MFK1-FA Kappabridge coupled with a CS3 heating apparatus at the Council for Geoscience, Pretoria, South Africa. Data were processed using Cureval(https://www.agico.com/text/software/cureval/cureval.php). Magnetic susceptibility versus temperature (κ-T) curves exhibits distinct characteristics for different magnetic carrier minerals and thus allow for their characterization and the identification of possible phase transitions during thermal demagnetization.

### Proterozoic carbonate platform paleolatitude compilation

We compiled the temporal variation of paleolatitude for neritic carbonate deposits during the Proterozoic Eon (2500–541 Ma) from published direct and indirect paleomagnetic results as listed in the Global Paleomagnetic Database or GPMD^24^ and from more recent publications that are not yet incorporated in the GPMD. The GPMD lists numerous entries from data catalogues particularly for units from the former Union of Soviet Socialist Republics and a few from unpublished PhD theses, such entries were not included in our compilation given the difficulty to asses data quality and lack of peer review. Inclusion in our compilation further requires a presumption that the age of magnetization is the same as the rock age and that the studied rock unit contains neritic carbonates. Note that the paleomagnetic data can be obtained directly from neritic carbonates (limestone or dolomite) or from other lithologies that are conformable with carbonate deposits within the same succession. A total of 2569 Proterozoic paleomagnetic studies were listed in the GPMD between 1953 and 2021. Those entries where the age of magnetization is suspected to be different from the rock age (i.e., entries are likely magnetic overprints) were discarded. Particular caution was applied to studies that pre-date 1980, as these pre-date the use of magnetic component characterization by least-squares analysis^52^. Superseded studies were excluded. Some duplicate entries were kept where studies were published independently but around the same time. We evaluated and report the R score^56^ for each entry in our compilation to screen in terms of quality. Because the age of rock units are not always updated in the GPDM as new constraints become available, each of the entries had to be reviewed to establish the most recent acceptable constraints. A total of 140 data entries appear in our final compilation (Supplementary Table 3). Where possible, the approximate stratigraphic thickness of carbonate is reported for each of these to provide a proxy for the volume of carbonate deposition.

### Supplementary information

Supplementary Information

Peer Review File

## Data Availability

The authors declare that data supporting the findings of this study are available within the paper and its supplementary information files.
