# Supplementary material for: High-latitude platform carbonate deposition constitutes a climate conundrum at the terminal Mesoproterozoic
Source: Nat Commun. 2024 Mar 6;15:2024. doi: 10.1038/s41467-024-46390-w (PMC10918070; doi:10.1038/s41467-024-46390-w)
Supplement: Supplementary file 1 — Supplementary Information [file 41467_2024_46390_MOESM1_ESM.docx]

**High-latitude platform carbonate deposition constitutes a climate conundrum at the terminal Mesoproterozoic**

**SUPPLEMENTRAY METHODS**

**
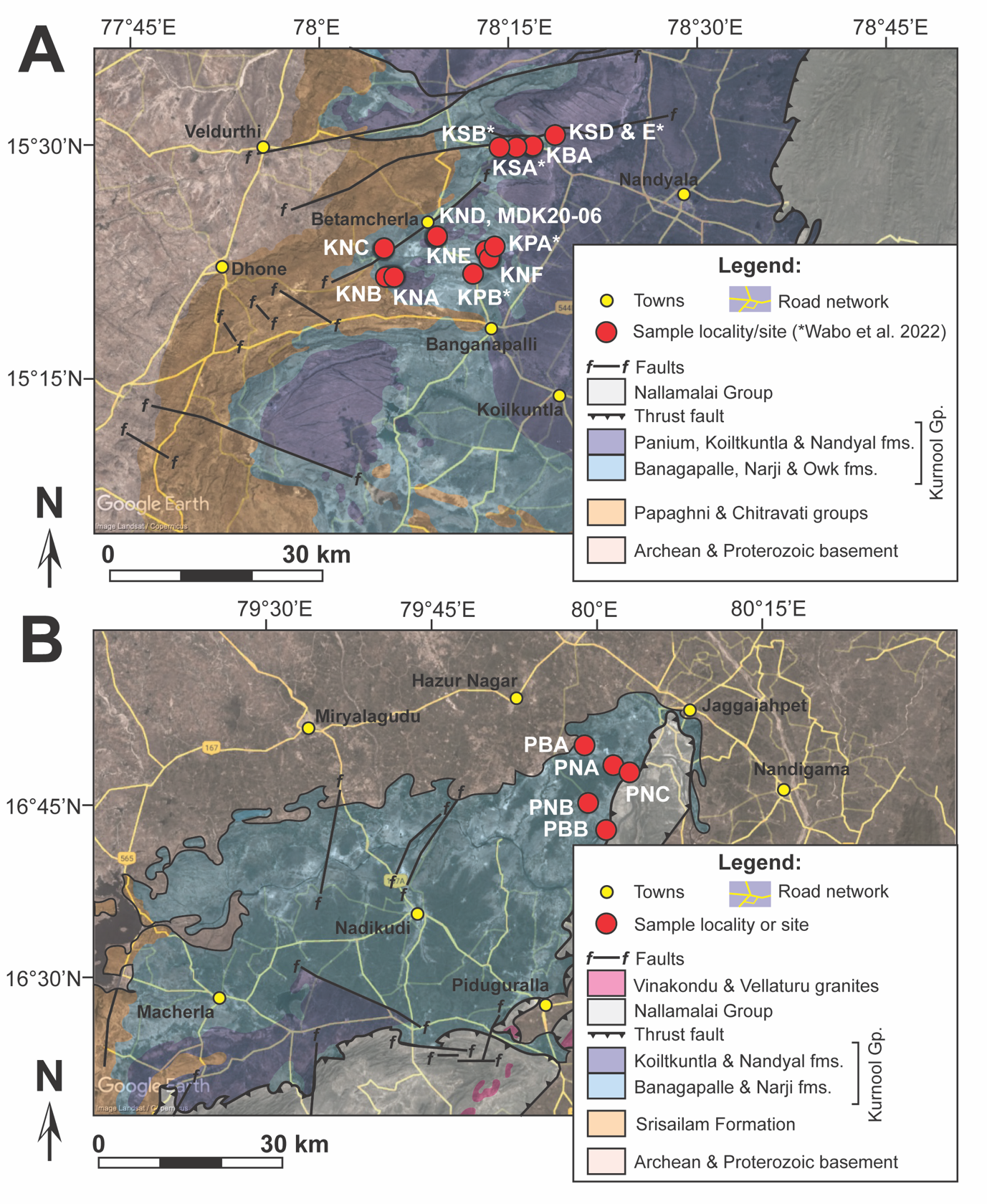
**

Supplementary Figure 1 **Simplified geology** **of the sampling areas**. **a** The Kurnool Basin. **b** The Palnad Basin. Geology after Radhakrishna^1^ are overlain onto satellite images with sampling localities of this and a previous study^2^

**SUPPLEMENTARY NOTES**

***Paleomagnetic results from the Banaganapalle Formation***

Only one of three sites yielded consistent magnetic components. Demagnetization results of samples from site KBA (Kurnool Basin) were somewhat noisy during low-field AF demagnetization and thermal demagnetization steps up to ~500°C, but remanence remained dominantly southeasterly and up throughout demagnetization. Southeast-up remanence components were revealed between 500°C to 640°C demagnetization steps (Supplementary Figure 2a, Supplementary Figure 4a, Supplementary Table 1) in all samples, except for KBA01 and 04, which had inconsistent demagnetization behavior. KBA01 is characterized by southeast and down remanence (Supplementary Figure 4a) and KBA04 behaved erratically during thermal demagnetization. Neither of the two sites from the Palnad Basin produced consistent magnetic components. No consistent low or high temperature remanence components could be identified for samples from sites PBA and PBB. Components had scattered distributions, and no means were calculated from these two sampling sites.

***Paleomagnetic results from the Narji Formation***

Samples from the Narji Formation are characterized by two magnetic components (Supplementary Figure 2 to 4). All sites yielded consistent components during demagnetization, except for sites PNA and PNC in the Palnad Basin where samples yielded scattered components. In six of our nine sites a shallow north and down remanence were identified during demagnetization up to ~200°C (Supplementary Figure 2 to 4, Supplementary Table 1). During higher temperature demagnetization steps, steep northerly-down or southerly-up components unblock above 600°C in pink to purplish limestone samples (i.e., sites KNA, KNB, and KND; Supplementary Figure 2 to 4, Supplementary Table 1), and at 420°C in grey to black limestone samples (i.e., KNC, KNE, KNF, and PNB; Supplementary Figure 2 to 4, Supplementary Table 1).

***Rockmagnetism***

Thermomagnetic or κ-T curves (Supplementary Figure 5) for pink calcareous shale, black limestone, and grey limestone (i.e., KND02 KNE01, PNA02, respectively) display relatively low initial magnetic susceptibilities. Magnetic susceptibility increases significantly after heating, with the magnetic susceptibility in cooling curves being considerably higher than that of heating curves at the same temperature below ~580°C. Above 580°C to 700°C the magnetic susceptibility of heating and cooling curves is reversable. KND02 and KNE01 display prominent decay of magnetic susceptibility at around ~580°C, while KND02 display an additional drop around 660°C. Sample KNE01 further display an increase in magnetic susceptibility between 400°C and 500°C during heating. A similar, but less pronounced increase is seen in KND02. These increases are likely due to the production of magnetite at the expense of iron-containing clay minerals. PNA02 has a very low initial magnetic susceptibility with an obvious drop around 400°C, and less prominent drops at 545°C and 580°C. These Curie temperatures suggest the presence of magnetite in all the samples, and both magnetite and hematite in sample KND02. The lower Curie temperature of 400°C in PNA02 could indicate the presence of an iron sulfide phase like pyrrhotite, but additional rock magnetic experiments would have to be conducted to confirm this.

**Supplementary Table 1. Summary of demagnetization results**

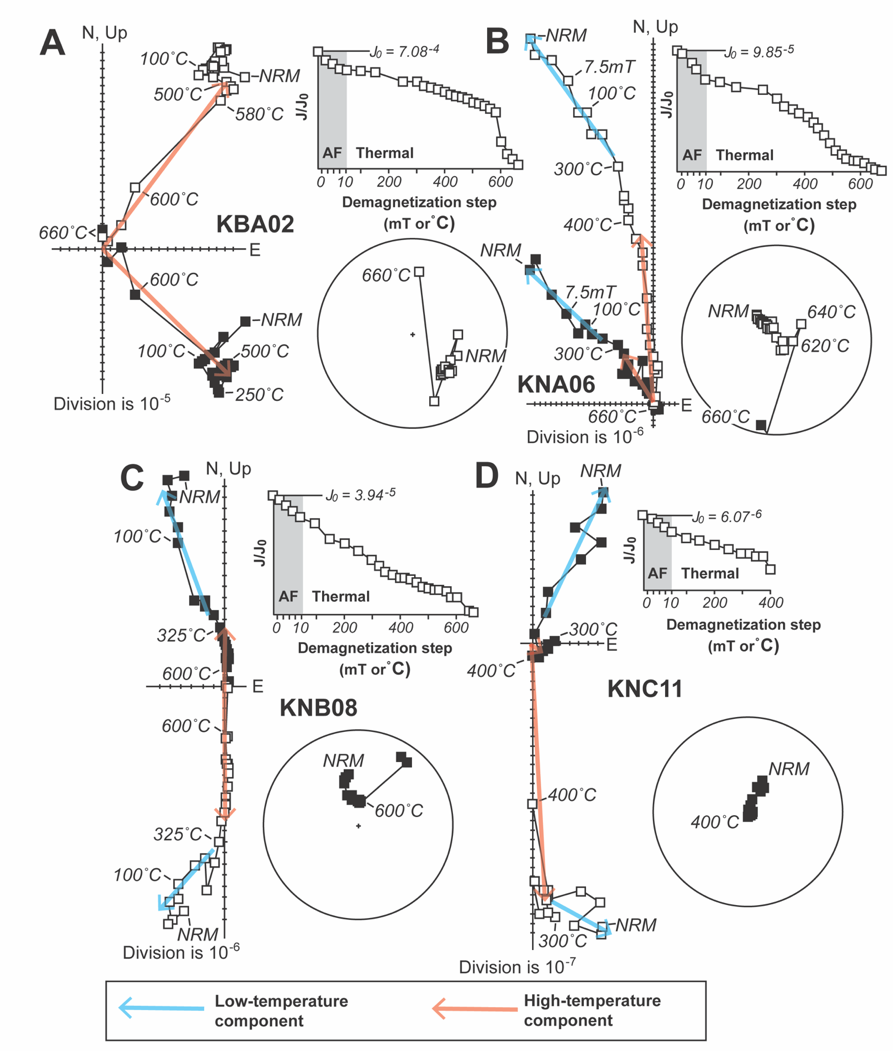


Supplementary Figure 2 **Representative demagnetization behavior.** Sample demagnetization from the Kurnool Basin is illustrated as orthogonal projections onto the horizontal and E-W vertical planes, as well as on equal area diagrams in geographic coordinates are shown together with normalized magnetization (J/Jo) decay plots. **a.** Banaganapalle Formation sandstone. **b and c.** Narji Formation pink limestone. **d.** Narji Formation grey limestone. Symbols on orthogonal plots: open = vertical projection, closed = horizontal projection. Symbols of equal area plots: open = upper hemisphere, closed = lower hemisphere. NRM = natural remanent magnetization.


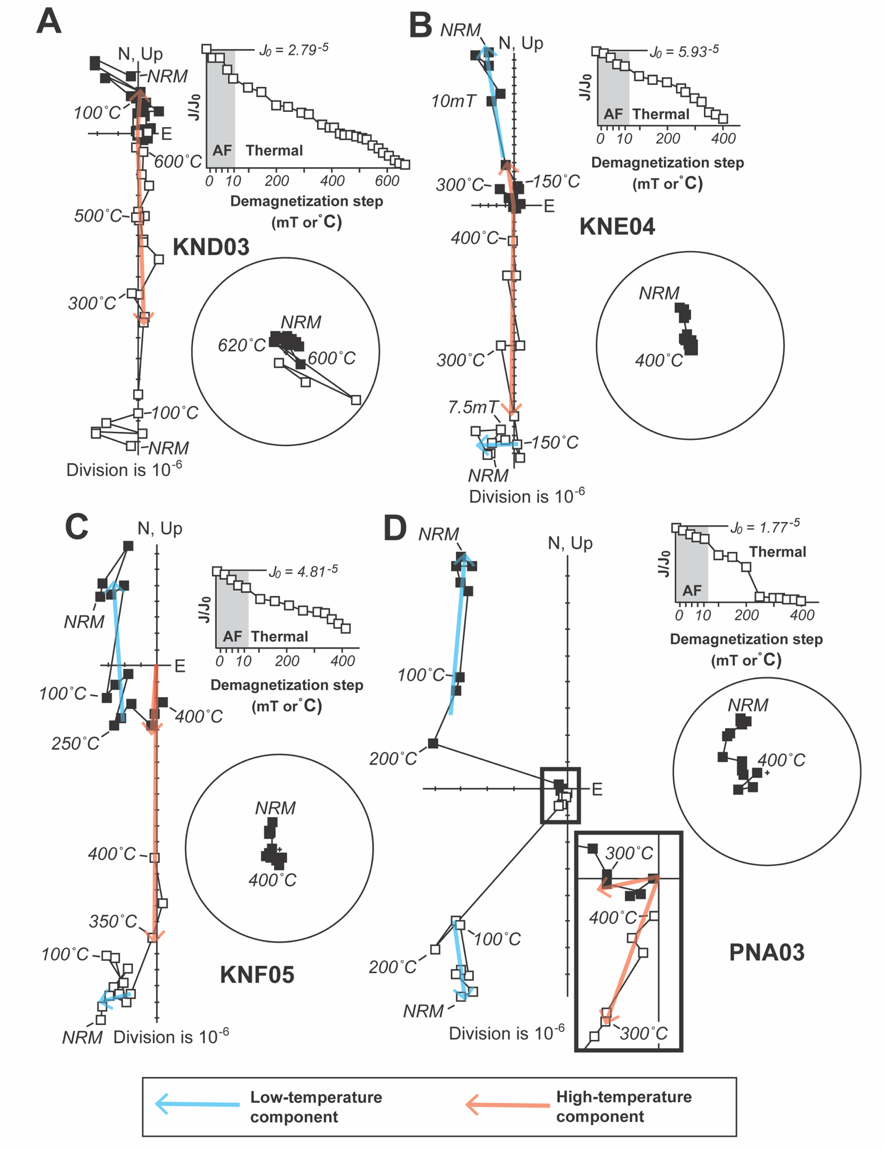


Supplementary Figure 3. **Representative demagnetization behavior.** Sample demagnetization from the Kurnool and Palnad basins are illustrated as orthogonal projections onto the horizontal and E-W vertical planes, as well as on equal area diagrams in geographic coordinates are shown together with normalized magnetization (J/Jo) decay plots. **a.** Narji Formation pink calcareous shale, Kurnool Basin. **b and c.** Narji Formation black limestone, Kurnool Basin. **d.** Narji Formation grey limestone, Palnad Basin. Symbols on orthogonal plots: open = vertical projection, closed = horizontal projection. Symbols of equal area plots: open = upper hemisphere, closed = lower hemisphere. NRM = natural remanent magnetization.

***
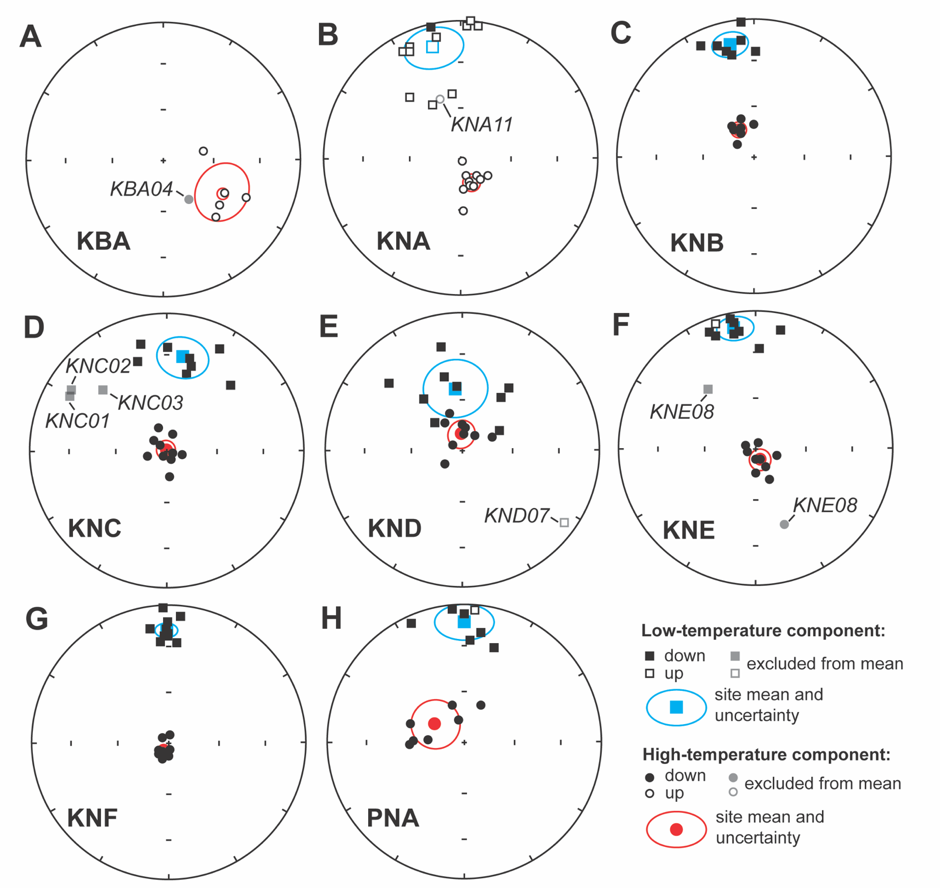
***

Supplementary Figure 4. **Summary of identified magnetic components.** In-situ low-temperature (i.e., 100-350°C) and high-temperature (i.e., 420-660°C) magnetic components are illustrated. **a.** Banaganapalle Formation sandstone, Kurnool Basin. **b and c.** Narji Formation pink limestone, Kurnool Basin.**d.** Narji Formation grey limestone, Kurnool Basin. **e.** Narji Formation pink calcareous shale, Kurnool Basin. **f and g.** Narji Formation black limestone, Kurnool Basin. **h.** Narji Formation grey limestone, Palnad Basin.


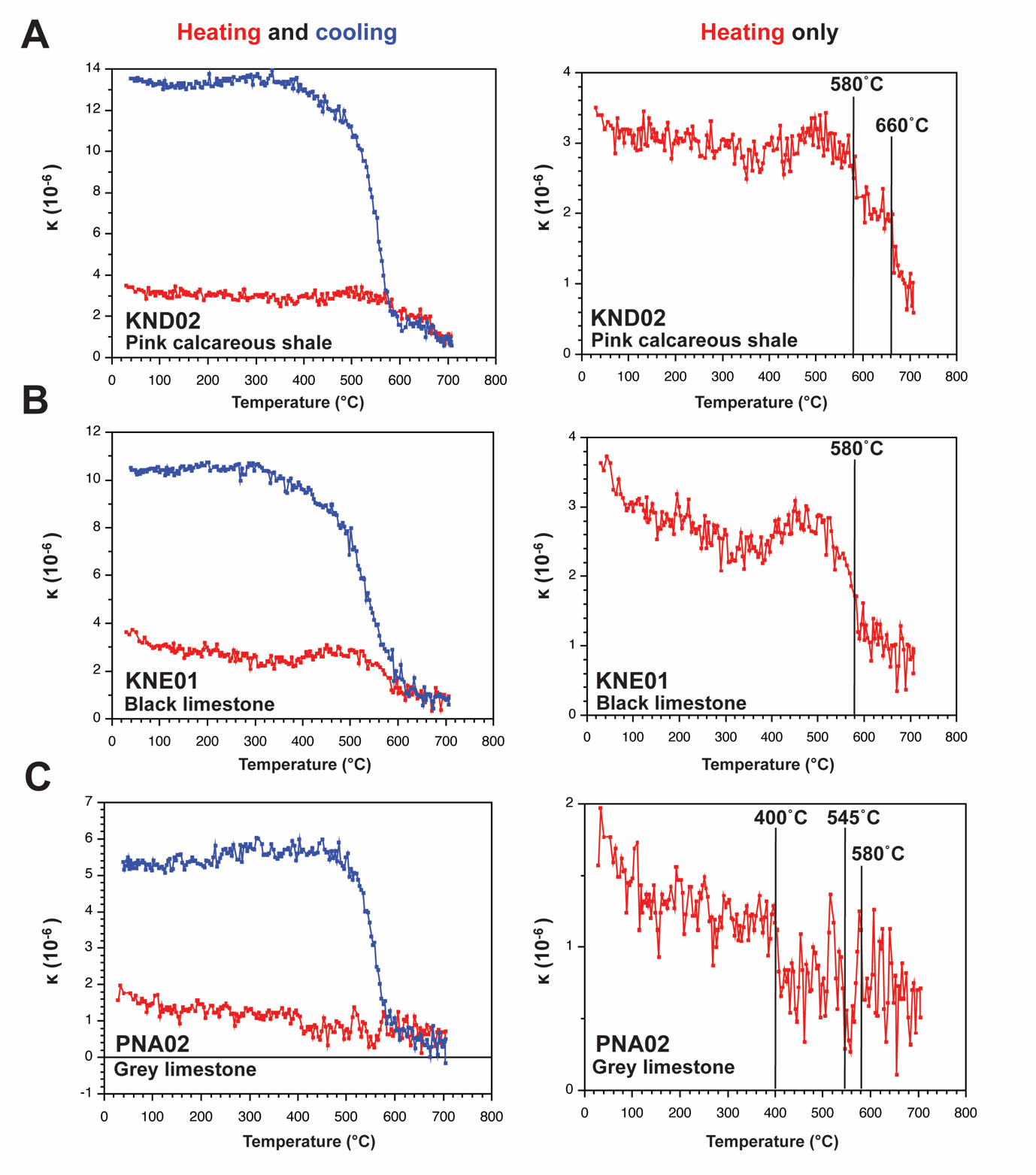


Supplementary Figure 5. **Representative thermomagnetic or κ-T curves from the Narji Formation.** **a.** Pink calcareous shale, Kurnool Basin. **b.** Black limestone, Kurnool Basin. **c.** Grey limestone, Palnad Basin.

***Comparison with published results***

Previous paleomagnetic constraints have been reported from the Kurnool Group^2,3^ and are here compared with our results (Supplementary Figure 6a and b).

A study by Goutham et al.^10^ reported paleomagnetic constraints from 23 sampling sites in the Banaganapalle (20 sites) and the Narji (3 sites) formations from the Kurnool and Palnad basins. Seventeen Banaganapalle Formation sites and two Narji Formation sites yielded (within-site) consistent demagnetization results. Unfortunately, site level data are not reported, but only mean directions (Supplementary Figure 6c). No information is provided on how data from multiple specimens from the same sample were handled during mean calculations. Apart from a few specimens that were subjected to pilot alternating field demagnetization most specimens were subjected to batch thermal demagnetization steps at 325°C, 425°C, 525°C, 600°C, 650°C, and 680°C. The Banaganapalle Formation yielded dual polarity and very shallow northerly and southerly means, while the Narji Limestone yielded a northerly shallow to moderately upward directed mean (Supplementary Figure 6c). No stability field tests were illustrated for these directions. It is noted that the shallow as well as shallow to moderately upwards northerly directions reported by Goutham et al.^10^ are very similar to low temperature magnetizations identified during the current study (Supplementary Figure 6a). No detailed illustration is provided for the demagnetization behavior of the Narji Formation specimens. The demagnetization behavior of only four specimens of the Banaganapalle Formation is illustrated as orthogonal plots, but directions are either steep southerly and upward directed or very steep northerly and downward and not near horizontal as reported (Supplementary Figure 6d). These steep directions are very similar to high-temperature magnetic components identified during the present study (Supplementary Figure 6a), but given the issues raised here around the study by Goutham et al.^10^, its results are best disregarded.

A study by Wabo et al.^2^ reported paleomagnetic constraints from six sites in the Narji Formation from the Kurnool Basin (Sites are shown in Supplementary Figure 1). Apart from a low stability component that is removed during very low temperature demagnetization steps (100°C to 350°C), a very shallow northerly magnetization was identified in three of the sampling sites between 300°C and 350°C (Supplementary Figure 6e), but this component is poorly constrained. At higher levels of demagnetization (up to 420°C to 460°C) very steep northerly down or southerly up magnetizations unblocked as high stability magnetic directions (Supplementary Figure 6f). Dispersed high stability components were isolated in 12 limestone clasts that otherwise carried consistent shallow north-down, and very shallow northerly lower stability magnetizations. Despite this seemingly positive outcome, Wabo et al.^2^ over cautiously interpreted it as indeterminate due to the elevated α_95_ value of 22.6° obtained for the high-temperature magnetization from the underlying parent lithology from which the clasts were likely sourced. Here we regard the test as positive for the high-temperature steep southerly magnetization and further note that the test is negative for the shallow north-down, and very shallow northerly magnetizations. Wabo et al.^2^ also illustrated a positive fold test for their high stability component, suggesting that it was acquired before regional deformation at ~1.0 Ga.

The similarity between the results of Wabo et al.^2^ and that of the present study is noted, but we did not identify the very shallow northerly magnetization between 300°C and 350°C. Low-temperature (i.e., 100°C to 350°C) components compare well, as do those identified at higher levels of demagnetization (Compare Supplementary Figure 6a and b with Supplementary Figure 6e and f). Two Narji Formation samples of Wabo et al.^2^, one of a black limestone (i.e., KPA07) and one of a grey limestone (i.e., KPB06) also yielded κ-T curves in the present study (Supplementary Figure 7) that are comparable to other samples from the area. That is, they are irreversible below 580°C and reversible between 580°C and 700°C. Curie temperatures identified at ~580°C suggest that magnetite is the dominant magnetic carrier. Sample KPB06 also display an increase in magnetic susceptibility between 400°C and 500°C during heating due to the possible growth of magnetite at the expense of iron-rich clay minerals. As such, the results of Wabo et al.^2^ are combined with those of the present study to calculate means of the components removed by low-temperature (i.e., 100°C to 350°C) and high-temperature (420-660°C) demagnetization of the Kurnool Group (Supplementary Table 1).

The steep high temperature components identified in the Banaganapalle and Narji Formations in this study and that of Wabo et al.^2^ are either northerly-up, or southerly-down. Together the data, however, do not conclusively pass reversal test. An angle between the means of λ = 21° is smaller than the critical angle, λ_c_ = 45° at which the means would be significantly different from another (‘Indeterminate’ class). This could be due to the small number of sites that record the southeast-up high-temperature component (n = 3) compared to those that record the northwest-down high-temperature component (n = 10).

**
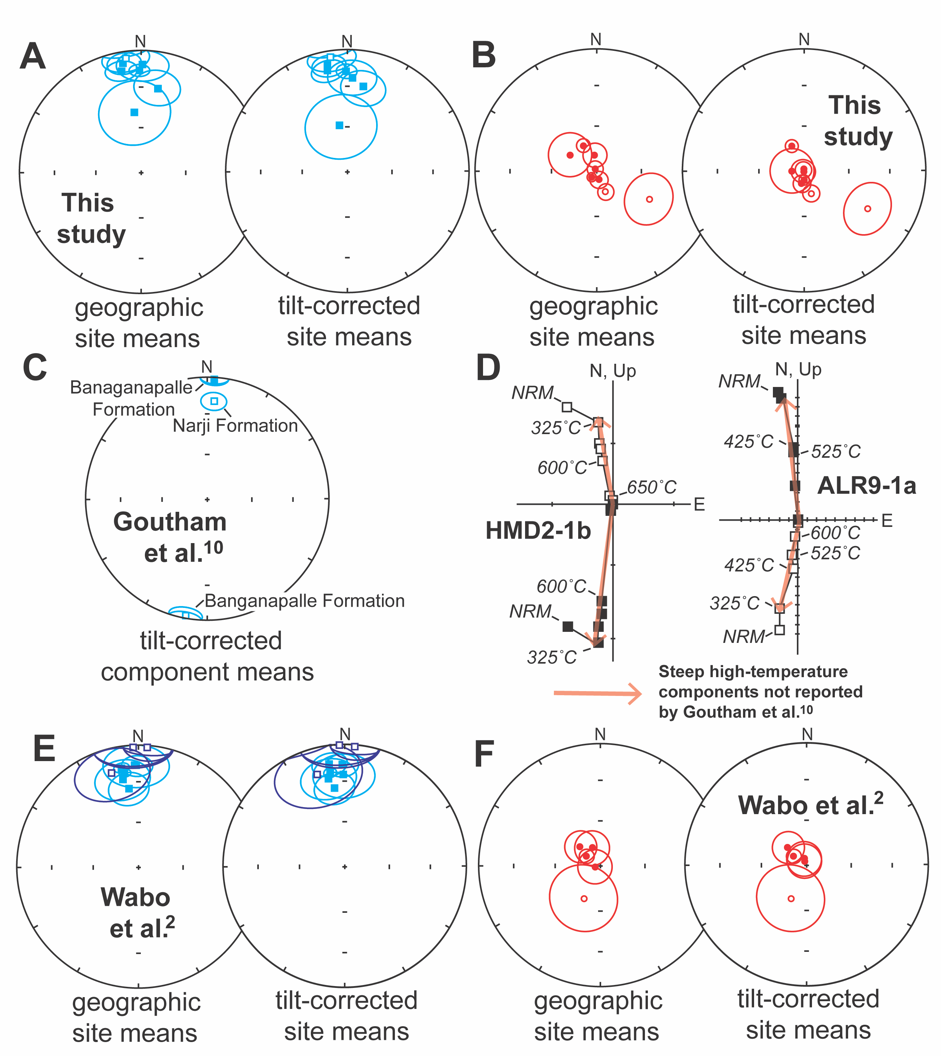
**

Supplementary Figure 6**. Summary of magnetic components identified from the Kurnool Group.** Equal area plot symbols: open = upper hemisphere, closed = lower hemisphere; Orthogonal plot symbols: open = vertical projection, closed = horizontal projection. **a.** Site means of low- temperature for components identified during this study. **b.** Site means of high-temperature components identified during this study. **c.** Component means reported by Goutham et al.^10^. **d.** Orthogonal plots of demagnetization behavior of two specimens from the Banaganapalle Formation (reproduced from Goutham et al.^10^). **e.** Site means of low- temperature for components identified by Wabo et al.^2^. Light blue symbols are components removed between 100°C and 350°C, and dark blue symbols are components removed between 300°C and 350°C. **F.** Site means of high-temperature components identified by Wabo et al.^2^.

**
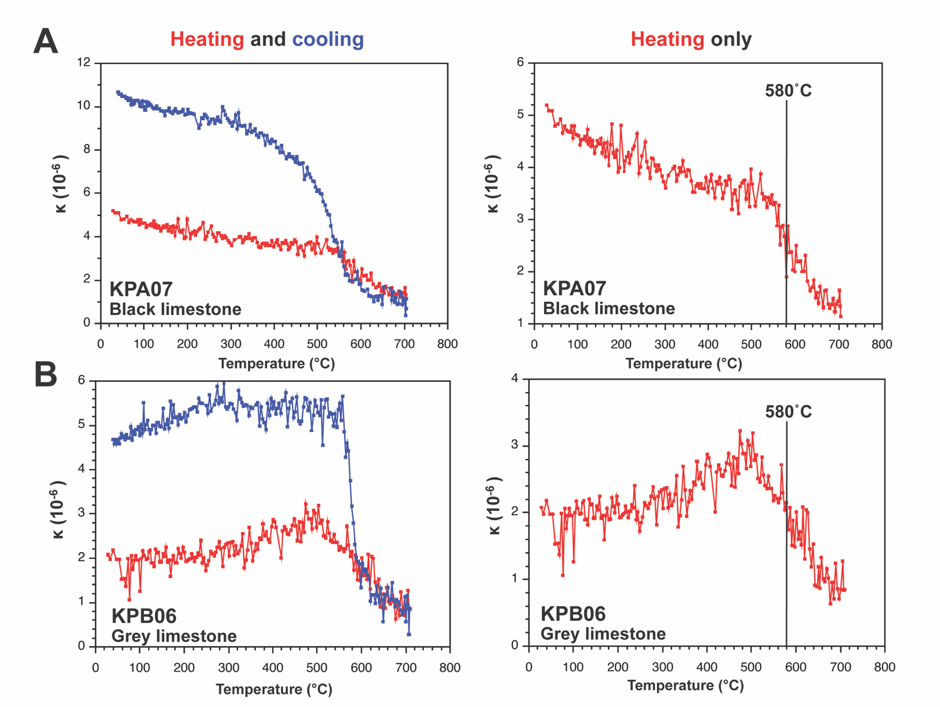
**

Supplementary Figure 7. **Thermomagnetic or κ-T curves for samples of Wabo et al.^2^ from the Narji Formation.** **a.** Black limestone, Kurnool Basin. **b.** Grey limestone, Kurnool Basin.

Together the data from this study and that of Wabo et al.^2^ yield a paleopole at 26.4°N and 66.6°E with A_95_ = 13.3° (Supplementary Figure 8). Our pole is different from the Phanerozoic apparent polar wander path of Gondwana when viewed in Indian coordinates^4^ (Supplementary Figure 8a), and when compared to published 1500-550 Ma paleomagnetic poles from India our pole differs from key paleomagnetic poles at 1465 Ma, 1075 Ma and 770 Ma^5^ (Supplementary Figure 8b). Our pole is similar to that of the 1192 ± 10 Ma Harohalli alkaline dykes^6^ (HAR in Supplementary Figure 8b). The Harohalli alkaline dykes pole was previously assigned an 800-850 Ma age based on Rb-Sr and K-Ar whole rock ages, but were reassigned as being 1192 Ma based on a U-Pb zircon age from one of the largest dykes of this swarm^6^. This pole, however, should be treated with some caution as the dated dyke did not yield paleomagnetic directions, and dykes that do have paleomagnetic constraints were not dated^6^. Apart from this, our pole is also similar to poles from limestone and shale from the Kaladgi and Bhima basins^7^, and the Chhattisgarh and Pranhita-Godavari Valley basins^8^ (K&B and C&PG in Supplementary Figure 8b), which represent the same stratigraphic sequence as the Kurnool Group (i.e., Sequence III). The pole for our low temperature magnetization is similar to directions previously reported from by Banaganapalle and Narji formations by reference 1 (BQ and NL in Supplementary Figure 8b)*.*

**
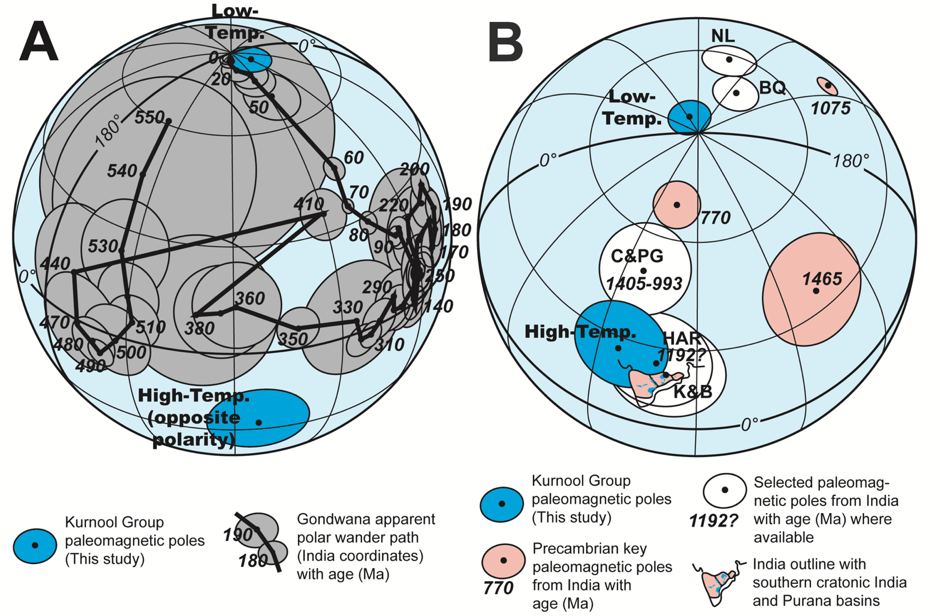
**

Supplementary Figure 8. **Kurnool Group paleomagnetic poles compared to published paleopoles.** **a**. Comparison with the Phanerozoic apparent polar wander path for Gondwana in Indian coordinates^4^. **B** Comparison with Precambrian key paleomagnetic poles from India^5^ and selected paleomagnetic poles from India (see Supplementary Table 2 for details).

**Supplementary Table 2. Summary of paleopoles used for comparison and reconstruction**

| **Craton:**  ***Unit*** | **Nominal age (Ma)** | **Pole latitude (°N)** | **Pole longitude (°E)** | **A95 or dp/dm (°)** | **Reference** |
| --- | --- | --- | --- | --- | --- |
| **Southern India:** |  |  |  |  |  |
| *Narji Limestone (NL)* | 0 | 62 | 263 | 7 | 3 |
| *Banaganapalle Formation (BQ)* | 0 | 74 | 234 | 5 | 3 |
| *Kurnool Group low-temperature component* | 0 | 85 | 298 | 6 | This study |
| *India 770 Ma key pole* | 770 | 69 | 74 | 6 | 5 |
| *India 1075 Ma key pole* | 1075 | 44 | 215 | 3 | 5 |
| *Harohalli alkaline dykes* | 1192 | 25 | 78 | 15 | 6 |
| *Kaladgi and Bhima basins (K&B)* | ~1200 | 22 | 81 | 16 | 7 |
| *Chhattisgarh and Pranhita-Godavari Valley basins (Ch-PG)* | ~1200 | 50 | 67 | 12 | 8 |
| *Kurnool Group high-temperature component* | ~1200 | 26 | 67 | 13 | This study |
| *South India 1465 Ma key pole* | 1465 | 36 | 132 | 16 | 5 |
| **North China:** |  |  |  |  |  |
| *Jingeryu Formation (#54)* | ~1100 | 67 | 132 | 23/26 | 9 |
| *Lower part of Nanfen Fm and equivalents (Xihe Group, Xinxing Fm, Liulaobei Fm)(#55)* | ~1100 | 39 | 137 | 3 | 10 |
| *Upper part of lower member of Nanfen Fm (Xihe Group, Benxi & Dalian region) (#56)* | ~1100 | 8 | 129 | 8 | 10 |
| *Middle member of Nanfen Fm (Xihe Group, Benxi & Dalian region) (#57)* | ~1100 | -11 | 128 | 9 | 10 |
| *Nanfen Formation (Xihe Group, Benxi region) (#58)* | ~1100 | 64 | 162 | 9/11 | 9 |
| *Liulaobei Formation (Huainan Group) (#59)* | ~1100 | 43 | 150 | 12 | 11 |

**^9-11^**

**^2,7-10,12-108^**

^10^

**Supplementary Table 3. Compilation of Precambrian carbonate platform paleolatitudes**

**Supplementary Table 3.** Continued

**Supplementary Table 3.** Continued

**Supplementary Table 3.** Continued

**SUPPLEMENTARY REFERENCES**

1 Radhakrishna, B. P. *Purana Basins of Peninsular India (Middle to Late Proterozoic)*. (Geological Society of India, Memoir 6, 1987).

2 Wabo, H. *et al.* Paleomagnetic and 40Ar/39Ar age constraints on the timing of deposition of deep-water carbonates of the Kurnool Group (Cuddapah basin) and correlation across Proterozoic Purana successions of Southern India. *J. Asian Earth Sci.* **223**, doi:10.1016/j.jseaes.2021.104984 (2022).

3 Goutham, M. R., Raghubabu, K., Prasad, C. V. R. K., Subba Rao, K. V. & Damodara Reddy, V. A Neoproterozoic geomagnetic field reversal from the Kurnool Group, India: Implications for stratigraphic correlation and formation of Gondwanaland. *Journal Geological Society of India* **67**, 221-233 (2006).

4 Torsvik, T. H. *et al.* Phanerozoic polar wander, palaeogeography and dynamics. *Earth-Science Reviews* **114**, 325-368, doi:10.1016/j.earscirev.2012.06.007 (2012).

5 Meert, J. G., Pivarunas, A. F., Miller, S. R., Pandit, M. K. & Sinha, A. K. in *Ancient Supercontinents and the Paleogeography of Earth* (eds L. J. Pesonen *et al.*) Ch. 10, 305-332 (Elsevier, 2021).

6 Pradhan, V. R., Pandit, M. K. & Meert, J. G. in *Indian Dykes* (ed Srivastava et al.) 339-352 (Narosa Publishing House, 2008).

7 Wabo, H., De Kock, M. O., Beukes, N. J. & Hedge, V. S. Palaeomagnetism of the uppermost carbonate units of the Purana basins in southern India: new demagnetization results from the Kaladgi and Bhima basins, Karnataka. *Geol. Mag.* **159**, 269-278 (2022).

8 De Kock, M. O., Beukes, N. J. & Mukhopadhyay, J. Palaeomagnetism of Mesoproterozoic limestone and shale successions of some Purana basins in southern India. *Geol. Mag.* **152**, 728-750 (2015).

9 Zhang, H. & Zhang, W. Palaeomagnetic data, Late Precambrian magnetostratigraphy and tectonic evolution of eastern China. *Precambrian Res.* **29**, 65-75 (1985).

10 Zhao, H. *et al.* New geochronologic and paleomagnetic results from early Neoproterozoic mafic sills and late Mesoproterozoic to early Neoproterozoic successions in the eastern North China Craton, and implications for the reconstruction of Rodinia. *GSA Bulletin* **132**, 739-766, doi:10.1130/b35198.1 (2020).

11 Piper, J. D. A. & Zhang, Q. R. Palaeomagnetism of Neoproterozoic glacial rocks of the Huabei Shield: the North China Block in Gondwana. *Tectonophysics* **283**, 145-171 (1997).

12 Swanson-Hysell, N. L., Killian, T. M. & Hanson, R. E. A new grand mean paleomagnetic pole for the Umkondo Igneous Province with implications for paleogeography and the geomagnetic field. *Geophys. J. Int.* **203**, 2237-2247 (2015).

13 Hanson, R. E. *et al.* Coeval large-scale magmatism in the Kalahari and Laurentian cratons during Rodinia assembly. *Science* **304**, 1126-1129 (2004).

14 Gumsley, A. P. *et al.* Timing and tempo of the Great Oxidation Event. *Proc. Nat. Acad. Sci. U.S.A.* **114**, 1811-1816, doi:10.1073/pnas.1608824114 (2017).

15 Martin, D. L., Nairn, A. E. M., Noltimier, H. C., Petty, M. H. & Schmitt, T. J. Paleozoic and Mesozoic paleomagnetic resukts from Marocco. *Tectonophysics* **44**, 91-114 (1978).

16 Landing, E. *et al.* Duration of the Early Cambrian U-Pb ages of volcanic ashes from Avalon and Gondwana. *Can. J. Earth Sci.* **35** (1998).

17 Boudzoumou, F., Vandamme, D., Affaton, P. & Gattacceca, J. Neoproterozoic paleomagnetic poles in the Taoudeni basin (West Africa). *C.R. Geosci.* **343**, 284-294, doi:10.1016/j.crte.2010.12.001 (2011).

18 Deynoux, M., Affaton, P., Trompette, R. & Villeneuve, M. Pan-African tectonic evolution and glacial events registered in Neoproterozoic to Cambrian cratonic and foreland basins of West Africa. *J. Afr. Earth Sci.* **46**, 397-426, doi:10.1016/j.jafrearsci.2006.08.005 (2006).

19 Perrin, M., Elston, D. P. & Moussine-Pouchkine, A. Paleomagnetism of Proterozoic and Cambrian strata, Adrar de Mauritanie, cratonic West Africa. *J. Geophys. Res.* **93**, 2159-2178, doi:10.1029/JB093iB03p02159 (1988).

20 Álvaro, J. J., Macouin, M., Bauluz, B., Clausen, S. & Ader, M. The Ediacaran sedimentary architecture and carbonate productivity in the Atar cliffs, Adrar, Mauritania: Palaeoenvironments, chemostratigraphy and diagenesis. *Precambrian Res.* **153**, 236-261, doi:10.1016/j.precamres.2006.11.010 (2007).

21 Rooney, A. D., Selby, D., Houzay, J.-P. & Renne, P. R. Re-Os geochronology of Mesoproterozoic sedimentary succession, Taoudeni basin, Mauritania: implications for basin-wide correlations and Re-Os organic-rich sediments systematics. *Earth Planet. Sci. Lett.* **289**, 486-496 (2010).

22 Klootwijk, C. T. Eartly Paleozoic Palaeomagnetism in Australia. I. Cambrian results from the Flinders Ranges, South Australia. II. Late Early Cambrian results from Kangaroo Island, South Australia. III. Middle to early-Late Cambrian results from the Amadeus Basin, Northern Territory. *Tectonophysics* **64**, 249-332 (1980).

23 Kirschvink, J. L. The Precambrian-Cambrian boundary problem: paleomagnetic directions from the Amadeus Basin, Central Australia. *Earth Planet. Sci. Lett.* **40**, 91-100 (1978).

24 Li, Z. X. New palaeomagnetic results from the ‘cap dolomite’ of the Neoproterozoic Walsh Tillite, northwestern Australia. *Precambrian Res.* **100**, 359-370 (2000).

25 Swanson-Hysell, N. L. *et al.* Constraints on Neoproterozoic paleogeography and Paleozoic orogenesis from paleomagnetic records of the Bitter Springs Formation, Amadeus Basin, central Australia. *Am. J. Sci.* **312**, 817-884, doi:10.2475/08.2012.01 (2012).

26 Idnurm, M. Towards a high resolution Late Palaeoproterozoic - earliest Mesoproterozoic apparent polar wander path for northern Australia. *Aust. J. Earth Sci.* **47**, 405-429 (2000).

27 Idnurm, M., Giddings, J. W. & Plumb, K. A. Apparent polar wander and reversal stratigraphy of the Palaeo-Mesoproterozoic southeastern McArthur Basin, Australia. *Precambrian Res.* **72**, 1-41 (1995).

28 Schmidt, P. W. & Williams, G. E. Palaeomagnetism of red beds from the Kimberley Group, Western Australia: Implications for the palaeogeography of the 1.8Ga King Leopold glaciation. *Precambrian Res.* **167**, 267-280, doi:10.1016/j.precamres.2008.09.002 (2008).

29 Schmidt, P. W. & Williams, G. A. Ediacaran palaeomagnetism and apparent polar wander path for Australia: no large true polar wander. *Geophys. J. Int.* **182**, 711-726, doi:10.1111/j.1365-246X.2010.04652.x (2010).

30 Schmidt, P. W., Williams, G. E. & McWilliams, M. O. Palaeomagnetism and magnetic anisotropy of late Neoproterozoic strata, South Australia: Implications for the palaeolatitude of late Cryogenian glaciation, cap carbonate and the Ediacaran System. *Precambrian Res.* **174**, 35-52, doi:10.1016/j.precamres.2009.06.002 (2009).

31 Williams, G. E. & Schmidt, P. W. Low paleolatitude for the late Cryogenian interglacial succession, South Australia: paleomagnetism of the Angepena Formation, Adelaide Geosyncline. *Aust. J. Earth Sci.* **62**, 243-253, doi:10.1080/08120099.2015.1003967 (2015).

32 Pisarevsky, S. A., Wingate, M. T. D., Stevens, M. K. & Haines, P. W. Palaeomagnetic results from the Lancer 1 stratigraphic drillhole, Officer Basin, Western Australia, and implications for Rodinia reconstructions. *Aust. J. Earth Sci.* **54**, 561-572, doi:10.1080/08120090701188962 (2007).

33 Williams, G. E., Schmidt, P. W. & Clark, D. A. Palaeomagnetism of iron-formation from the late Palaeoproterozoic Frere Formation, Earaheedy Basin, Western Australia: palaeogeographic and tectonic implications. *Precambrian Res.* **128**, 367-383 (2004).

34 Rapalini, A. E., Trindade, R. I. & Poiré, D. G. The La Tinta pole revisited: Paleomagnetism of the Neoproterozoic Sierras Bayas Group (Argentina) and its implications for Gondwana and Rodinia. *Precambrian Res.* **224**, 51-70, doi:10.1016/j.precamres.2012.09.007 (2013).

35 Trindade, R. I. F., D’Agrella-Filho, M. S., Babinski, M., Font, E. & Brito Neves, B. B. Paleomagnetism and geochronology of the Bebedouro cap carbonate: evidence for continental-scale Cambrian remagnetization in the São Francisco craton, Brazil. *Precambrian Res.* **128**, 83-103, doi:10.1016/j.precamres.2003.08.010 (2004).

36 Trindade, R. I. F., Font, E., D'Agrella-Filho, M. S., Nogueira, A. C. R. & Riccomini, C. Low-latitude and multiple geomagnetic reversals in the Neoproterozoic Puga cap carbonate, Amazon craton. *Terra Nova* **15**, 441-446, doi:10.1046/j.1365-3121.2003.00510.x (2003).

37 Santos, R. F. d., Nogueira, A. C. R., Romero, G. R., Soares, J. L. & Bandeira Junior, J. Life in the aftermath of Marinoan glaciation: The giant stromatolite evolution in the Puga cap carbonate, southern Amazon Craton, Brazil. *Precambrian Res.* **354**, doi:10.1016/j.precamres.2020.106059 (2021).

38 Rapalini, A. E. & Astini, R. A. Paleomagnetic confirmation of the Laurentian origin of the Argentine Precordillera. *Earth Planet. Sci. Lett.* **155**, 1-14 (1998).

39 Maloof, A. C. *et al.* Combined paleomagnetic, isotopic, and stratigraphic evidence for true polar wander from the Neoproterozoic Akademikerbreen Group, Svalbard, Norway. *Geol. Soc. Am. Bull.* **118**, 1099-1124 (2006).

40 Lubnina, N. V., Pisarevsky, S. A., Puchkov, V. N., Kozlov, V. I. & Sergeeva, N. D. New paleomagnetic data from Late Neoproterozoic sedimentary successions in Southern Urals, Russia: implications for the Late Neoproterozoic paleogeography of the Iapetan realm. *Int. J. Earth Sci.* **103**, 1317-1334, doi:10.1007/s00531-014-1013-x (2014).

41 Bylund, G. Palaeomagnetism of Vendian-Early Cambrian sedimentary rocks from E Finnmark, Norway. *Tectonophysics* **231**, 45-57 (1994).

42 Pavlov, V. & Gallet, Y. Variations in geomagnetic reversal frequency during the Earth's middle age. *Geochem. Geophys. Geosyst.* **11**, 1-28, doi:10.1029/2009gc002583 (2010).

43 Urrutia-Fucugauchi, J. & Tarling, D. H. Palaeomagnetic properties of Eocambrian sediments in northwestern Scotland: implications for world-wide glaciation in the late Precambrian. *Palaeogeog. Palaeoclimatol. Palaeoecol.* **41**, 325-344 (1983).

44 Strachan, R. A. & Woodcock, N. H. in *Encyclopedia of Geology* 328-337 (2021).

45 Hirt, A. M., Lowrie, W., Julivert, M. & Arboleya, M. L. Paleomagnetic results in support of a model for the origin of the Asturian arc. *Tectonophysics* **213**, 321-339 (1992).

46 Kempf, O., Kellerhals, P., Lowrie, W. & Matter, A. Paleomagnetic directions in Late Precambrian glaciomarine sedimens of the Mirbat Sandstone Formation, Oman. *Earth Planet. Sci. Lett.* **175**, 181-190 (2000).

47 Allen, P. A. The Huqf Supergroup of Oman: Basin development and context for Neoproterozoic glaciation. *Earth-Science Reviews* **84**, 139-185, doi:10.1016/j.earscirev.2007.06.005 (2007).

48 Zhang, S., Li, Z. X., Wu, H. & Wang, H. New paleomagnetic results from the Neoproterozoic successions in southern North China Block and paleogeographic implications. *Science in China (Series D)* **43**, 233-243 (2000).

49 Zhao, X., Coe, R. S., Liu, C. & Zhou, Y. New Cambrian and Ordovician paleomagnetic poles for the North China Block and their paleogeographic implications. *Journal of Geophysical Research: Solid Earth* **97**, 1767-1788, doi:10.1029/91jb02742 (1992).

50 Huang, B., Yang, Z., Otofuji, Y. & Zhu, R. Early Paleozoic paleomagnetic poles from the western part of the North China Block and their implications. *Tectonophysics* **308**, 377-402 (1999).

51 Zhang, S., Li, Z. X. & Wu, H. New Precambrian paleomagnetic constraints on the position of the North China Block in Rodinia. *Precambrian Res.* **144**, 213-238 (2006).

52 Fu, X. *et al.* New paleomagnetic results from the Huaibei Group and Neoproterozoic mafic sills in the North China Craton and their paleogeographic implications. *Precambrian Res.* **269**, 90-106, doi:10.1016/j.precamres.2015.08.013 (2015).

53 Zhao, H. *et al.* New paleomagnetic results from the ca. 1.0 Ga Jiayuan Formation of the Huaibei Group in the North China craton, and their paleogeographic implications. *Precambrian Res.* **379**, doi:10.1016/j.precamres.2022.106807 (2022).

54 Pei, J., Yang, Z. & Zhao, Y. A Mesoproterozoic paleomagnetic pole from the Yangzhuang Formation, North China and its tectonics implications. *Precambrian Res.* **151**, 1-13, doi:10.1016/j.precamres.2006.06.001 (2006).

55 Wu, H. New paleomagnetic results from the Yangzhuang Formation of the Jixian System, North China, and tectonic implications. *Chin. Sci. Bull.* **50**, doi:10.1360/982005-809 (2005).

56 Zhu, R. *et al.* Paleomagnetic constraints on the tectonic history of the major blocks of China during the Phanerozoic. *Science in China (Series D)* **41**, 1-16 (1998).

57 Wu, H. *et al.* Paleomagnetic results of Paleozoic and Meosozoic rocks from Xingshan-Zingui section in Hubei Province, South China. *Science in China (Series D)* **42**, 181-194 (1999).

58 Lin, J. L., Fuller, M. & Zhang, W. Y. Paleogeography of the North and South China blocks during the Cambrian. *J. Geodyn.* **2**, 91-114 (1985).

59 Wang, X.-P. *et al.* Dickinsonia from the Ediacaran Dengying Formation in the Yangtze Gorges area, South China. *Palaeoworld* **30**, 602-609, doi:10.1016/j.palwor.2021.01.002 (2021).

60 Zhang, S. *et al.* New paleomagnetic results from the Ediacaran Doushantuo Formation in South China and their paleogeographic implications. *Precambrian Res.* **259**, 130-142, doi:10.1016/j.precamres.2014.09.018 (2015).

61 Jing, X., Yang, Z., Tong, Y., Wang, H. & Xu, Y. Identification of multiple magnetizations of the Ediacaran strata in South China. *Geophys. J. Int.* **212**, 54-75, doi:10.1093/gji/ggx396 (2018).

62 Xian, H., Zhang, S., Li, H., Yang, T. & Wu, H. Geochronological and palaeomagnetic investigation of the Madiyi Formation, lower Banxi Group, South China: Implications for Rodinia reconstruction. *Precambrian Res.* **336**, doi:10.1016/j.precamres.2019.105494 (2020).

63 Davis, J. K., Meert, J. G. & Pandit, M. K. Paleomagnetic analysis of the Marwar Supergroup, Rajasthan, India and proposed interbasinal correlations. *J. Asian Earth Sci.* **91**, 339-351, doi:10.1016/j.jseaes.2013.09.027 (2014).

64 George, B. G. & Ray, J. S. Provenance of sediments in the Marwar Supergroup, Rajasthan, India: Implications for basin evolution and Neoproterozoic global events. *J. Asian Earth Sci.* **147**, 254-270, doi:10.1016/j.jseaes.2017.07.027 (2017).

65 Malone, S. J. *et al.* Paleomagnetism and detrital zircon geochronology of the Upper Vindhyan sequence, Son Valley and Rajastan, India: A ca. 1000Ma closure age for the Purana Basins? *Precambrian Res.* **164**, 137-159 (2008).

66 Parashuramulu, V., Shankar, R., Srinivasa Sarma, D., Nagaraju, E. & Ramesh Babu, N. Baddeleyite Pb-Pb geochronology and paleomagnetic poles for ~1.89-1.86 Ga mafic intrusions from the Dharwar craton, India, and their paleogeographic implications. *Tectonophysics* **805**, 228789, doi:10.1016/j.tecto.2021.228789 (2021).

67 Kirschvink, J. L. & Rozanov, A. Y. Magnetostratigraphy of lower Cambrian strata from the Siberian Platform: a palaeomagnetic pole and a preliminary polarity time scale. *Geol. Mag.* **121**, 189-203 (1984).

68 Pavlov, V. E., Gallet, Y. & Shatsillo, A. V. Paleomagnetism of the Upper Riphean Lakhandinskaya Group in the Uchuro-Maiskii area and the hypothesis of the late Proterozoic supercontinent. *Izvestiya Phys. Solid Earth* **36**, 23-34 (2000).

69 Gallet, Y., Pavlov, V. E., Semikhatov, M. A. & Petrov, P. Y. Late Mesoproterozoic magnetostratigraphic results from Siberia: Paleogeographic implications and magnetic field behavior. *Journal of Geophysical Research: Solid Earth* **105**, 16481-16499, doi:10.1029/1999jb900354 (2000).

70 Pavlov, V. E., Gallet, Y., Shatsillo, A. V. & Vodovozov, V. Y. Paleomagnetism of the Lower Cambrian from the lower Lena River Valley: Constraints on the apparent polar wander path from the Siberian Platform and the anomalous behavior of the geomagnetic field at the begining of the Phanerozoic. *Izvestiya Phys. Solid Earth* **40**, 114-133 (2004).

71 Gurevitch, E. L. Paleomagnetic investigation of Precambrian deposits of the northern Siberian platform. **Paleomagnetizm verkhnego dokembriya (Trudy VNIGRI, Leningrad)**, 39-51 (1983).

72 Komissarova, R. A. Paleomagnetism of Precambrian rocks in the northern and eastern surroundings of the Siberian Platform. *Paleomagnetism and Geodynamics of the USSR territory, VNIGRI, Leningrad*, 38 (1991).

73 Pavlov, V. E., Gallet, Y. & Petrov, P. Y. A new Siberian record of the ∼1.0 Gyr-old Maya superchron. *Precambrian Res.* **320**, 350-370, doi:10.1016/j.precamres.2018.11.005 (2019).

74 Gallet, Y., Pavlov, V., Halverson, G. & Hulot, G. Toward constraining the long-term reversing behavior of the geodynamo: A new “Maya” superchron ∼1 billion years ago from the magnetostratigraphy of the Kartochka Formation (southwestern Siberia). *Earth Planet. Sci. Lett.* **339-340**, 117-126, doi:10.1016/j.epsl.2012.04.049 (2012).

75 Wingate, M. T. D. *et al.* Geochronology and paleomagnetism of mafic igneous rocks in the Olenek Uplift, northern Siberia: Implications for Mesoproterozoic supercontinents and paleogeography. *Precambrian Res.* **170**, 256-266, doi:10.1016/j.precamres.2009.01.004 (2009).

76 Evans, D. A. D., Veselovsky, R. V., Petrov, P. Y., Shatsillo, A. V. & Pavlov, V. E. Paleomagnetism of Mesoproterozoic margins of the Anabar Shield: A hypothesized billion-year partnership of Siberia and northern Laurentia. *Precambrian Res.* **281**, 639-655, doi:10.1016/j.precamres.2016.06.017 (2016).

77 Pavlov, V. E., Tkachenko, V. I. & Bondarenko, G. E. Palaeolatitudal position of the Western Pre-Kolyma block in the middle and late Riphean. *Doklady Akademii Nauk Rossii* **340**, 229-233 (1995).

78 Wen, B., Evans, D. A. D. & Li, Y.-X. Neoproterozoic paleogeography of the Tarim Block: An extended or alternative “missing-link” model for Rodinia? *Earth Planet. Sci. Lett.* **458**, 92-106, doi:10.1016/j.epsl.2016.10.030 (2017).

79 Zhao, P., Chen, Y., Zhan, S., Xu, B. & Faure, M. The Apparent Polar Wander Path of the Tarim block (NW China) since the Neoproterozoic and its implications for a long-term Tarim–Australia connection. *Precambrian Res.* **242**, 39-57, doi:10.1016/j.precamres.2013.12.009 (2014).

80 Ren, R., Guan, S.-W., Zhang, S.-C., Wu, L. & Zhang, H.-Y. How did the peripheral subduction drive the Rodinia breakup: Constraints from the Neoproterozoic tectonic process in the northern Tarim Craton. *Precambrian Res.* **339**, doi:10.1016/j.precamres.2020.105612 (2020).

81 Bretshtein, Y. S., Gurary, G. Z. & Perchersky, D. M. Paleomagnetism of Paleozoic terrain rocks, South-Western Primorie. *Geology of the Pacific Ocean* **16**, 41-63 (1997).

82 Klootwijk, C. T., Nazirullah, R. & de Jong, K. A. Palaeomagnetic constraints on formation of the Mianwali reentrant, Trans-Indus and western Salt Range, Pakistan. *Earth Planet. Sci. Lett.* **80**, 394-414 (1986).

83 Bretshtein, Y. S., Gurary, G. Z. & Klimova, A. V. Preliminary paleomagnetic features of the early Paleozoic terrain complexes of the S-E Primorye. *Paleomagnetism and rock magnetism. Moscow, OIFZ RAN*, 13-15 (1996).

84 Kravchinsky, V. A., Konstantinov, K. M. & Cogné, J.-P. Palaeomagnetic study of Vendian and Early Cambrian rocks of South Siberia and Central Mongolia: was the Siberian platform assembled at this time? *Precambrian Res.* **110**, 61-92 (2001).

85 Kravchinsky, V. A., Sklyarov, E. V., Gladkochub, D. P. & Harbert, W. P. Paleomagnetism of the Precambrian Eastern Sayan rocks: Implications for the Ediacaran–Early Cambrian paleogeography of the Tuva-Mongolian composite terrane. *Tectonophysics* **486**, 65-80, doi:10.1016/j.tecto.2010.02.010 (2010).

86 Watts, D. R., Van der Voo, R. & French, R. B. Paleomagnetic investigation of the Cambrian Waynesboro and Rome Formations of the Valley and Ridge Province of the Appalachian Mountains. *J. Geophys. Res.* **85**, doi:10.1029/JB085iB10p05331 (1980).

87 Gillett, S. L. & Van Alstine, D. R. Paleomagnetism of Lower and Middle Cambrian sedimentary rocks from the Desert Range, Nevada. *J. Geophys. Res.* **84**, 4475-4489, doi:10.1029/JB084iB09p04475 (1979).

88 Park, J. K. Paleomagnetism of the late Neoproterozoic Blueflower and Risky formations of the northern Cordillera, Canada. *Can. J. Earth Sci.* **32**, 718-729 (1995).

89 Kodama, K. P. Combined magnetostratigraphy from three localities of the Rainstorm Member of the Johnnie Formation in California and Nevada, United States calibrated by cyclostratigraphy: A 13R/Ma reversal frequency for the Ediacaran. *Frontiers in Earth Science*, doi:10.3389/feat.2021.764714 (2021).

90 Park, J. K. & Jefferson, C. W. Magnetic and tectonic history of the Late Proterozoic Upper Little Dal and Coates Lake Groups of northwestern Canada. *Precambrian Res.* **52**, 1-35 (1991).

91 Eyster, A., Weiss, B. P., Karlstrom, K. & Macdonald, F. A. Paleomagnetism of the Chuar Group and evaluation of the late Tonian Laurentian apparent polar wander path with implications for the makeup and breakup of Rodinia. *GSA Bulletin* **132**, 710-738, doi:10.1130/b32012.1 (2020).

92 Weil, A. B., Geissman, J. W. & Voo, R. V. d. Paleomagnetism of the Neoproterozoic Chuar Group, Grand Canyon Supergroup, Arizona: implications for Laurentia’s Neoproterozoic APWP and Rodinia break-up. *Precambrian Res.* **129**, 71-92, doi:10.1016/j.precamres.2003.09.016 (2004).

93 Jefferson, C. W. & Parrish, R. R. Late Proterozoic stratigraphy, U–Pb zircon ages, and rift tectonics, Mackenzie Mountains, northwestern Canada. *Can. J. Earth Sci.* **26**, 1787-1801, doi:10.1139/e89-151 (1989).

94 Park, J. K. Paleomagnetism of the Mudcracked formation of the Precambrian Little Dal Group, Mackenzie Mountains, Northwest Territories, Canada. *Can. J. Earth Sci.* **21**, 371-375 (1984).

95 Park, J. K. Paleomagnetism of the Late Proterozoic sills in the Tsezotene Formation, Mackenzie Mountains, Northwest Territories, Canada. *Can. J. Earth Sci.* **18**, 1572-1580 (1981).

96 Palmer, H. C., Baragar, W. R. A., Fortier, M. & Foster, J. H. Paleomagnetism of Late Proterozoic rocks, Victoria Island, Northwest Territories, Canada. *Can. J. Earth Sci.* **20**, 1456-1469 (1983).

97 Park, J. K. in *Geological Survey of Canada Paper 92-1C Current Research, Part C, Candian Shield* 43-52 (Geological Survey of Canada, 1992).

98 Park, J. K. & Aitken, J. D. Paleomagnetism of the late Proterozoic Tsezotene Formation of Northwestern Canada. *J. Geophys. Res.* **91**, 4955-4970, doi:10.1029/JB091iB05p04955 (1986).

99 Turner, E. C. & Long, D. G. F. Basin architecture and syndepositional fault activity during deposition of the Neoproterozoic Mackenzie Mountains supergroup, Northwest Territories, CanadaNorthwest Territories Geoscience Office Contribution 0040. *Can. J. Earth Sci.* **45**, 1159-1184, doi:10.1139/e08-062 (2008).

100 Park, J. K. & Aitken, J. D. Paleomagnetism of the Katherine Group in the Mackenzie Mountains: implications for post-Grenville (Hadrynian) apparent polar wander. *Can. J. Earth Sci.* **23**, 308-323 (1986).

101 Morris, W. A. & McMechan, M. E. Paleomagnetism of the Middle Proterozoic Mount Nelson Formation: evidence for a regional remagnetization event in the Late Precambrian of the Cordillera. *Can. J. Earth Sci.* **20**, 561-567 (1983).

102 Cox, A. & Doell, R. R. Review of paleomagnetism. *Bull. Geol. Soc. Am.* **71**, 645-768 (1960).

103 Timmons, J. M. *et al.* Tectonic inferences from the ca. 1255–1100 Ma Unkar Group and Nankoweap Formation, Grand Canyon: Intracratonic deformation and basin formation during protracted Grenville orogenesis. *Geol. Soc. Am. Bull.* **117**, doi:10.1130/b25538.1 (2005).

104 Books, K. G. Paleomagnetism of some Lake Superior Keweenawan Rocks. *Geol. Surv. Prof. Pap.* **760**, 1-42 (1972).

105 Elston, D. P., Enkin, R. J., Baker, J. & Kisileskey, D. K. Tightening the Belt: Paleomagnetic-stratigraphic constraints on deposition, correlation, and deformation of the Middle Proterozoic (ca. 1.4 Ga) Belt-Purcell Supergroup, United States and Canada. *GSA Bulletin* **114**, 619-638 (2002).

106 Swanson-Hysell, N. L. in *Ancient Supercontinents and the Paleogeography of the Earth* (eds L.J. Pesonen *et al.*) (Elsevier, 2021).

107 Irving, E. & McGlynn, J. C. Palaeomagnetism in the Coronation Geosyncline and arrangement of continents in the middle Proterozoic. *Geophys. J. Roy. Astron. Soc.* **58**, 309-339, doi:10.1111/j.1365-246x.1979.tb0127.x (1979).

108 Zhao, H. *et al.* in *EGU* (Vienna, 2023).
