# Supplementary material for: High-latitude platform carbonate deposition constitutes a climate conundrum at the terminal Mesoproterozoic
Source: Nat Commun. 2024 Mar 6;15:2024. doi: 10.1038/s41467-024-46390-w (PMC10918070; doi:10.1038/s41467-024-46390-w)
Supplement: Supplementary file 2 — Peer Review File [file 41467_2024_46390_MOESM2_ESM.pdf]

nature portfolio

Peer Review File

High-latitude platform carbonate deposition constitutes a climate conundrum at the terminal Mesoproterozoic

Open Access This file is licensed under a Creative Commons Attribution 4.0 International License, which permits use, sharing, adaptation, distribution and reproduction in any medium or format, as long as you give appropriate credit to the original author(s) and the source, provide a link to the Creative Commons license, and indicate if changes were made. In the cases where the authors are anonymous, such as is the case for the reports of anonymous peer reviewers, author attribution should be to 'Anonymous Referee' followed by a clear attribution to the source work. The images or other third party material in this file are included in the article's Creative Commons license, unless indicated otherwise in a credit line to the material. If material is not included in the article's Creative Commons license and your intended use is not permitted by statutory regulation or exceeds the permitted use, you will need to obtain permission directly from the copyright holder. To view a copy of this license, visit http://creativecommons.org/licenses/by/4.0/.

Editorial Note: Parts of this Peer Review File have been redacted as indicated to remove third-party material where no permission to publish could be obtained.

REVIEWER COMMENTS

Reviewer #1 (Remarks to the Author):

First, I would like to thank the editors for the opportunity to read this interesting manuscript. I have been overcommitted recently, but I could not turn down a manuscript with such an interesting title.

Second, I would like to commend the authors on an interesting idea. You will see, below, that I do not think this work is ready for publication, but it is an intriguing idea and one that is innovative and has not been argued before.

Third, I have made extensive comments in the PDF of the manuscript. Please use these line comments during any revisions that you might make. I will only report the critical elements that lead to a decision of "not yet publishable" below:

(1) The first critical issue is the assertion that high-latitude carbonate deposition requires globally high temperatures. This is really the crux of the manuscript, but the hypothesis is not well supported (rather, it is simply asserted).

The absence of glacial activity during this time does not require dramatically higher temperatures. Polar glaciation also requires the existence of sufficient continental mass as reservoirs for ice (i.e. like Antarctica), or sufficient continental mass that restricts active flow of oceanic currents to higher latitudes (i.e. like the northern polar ocean). At times where there is little polar continental mass, oceans tend to not form ice, and are generally warmer, but need not be drastically so.

(2) The second critical issue is the assertion that warmer temperatures (see above) is the uniformitarian approach. In fact, it is not. On the modern Earth, we have higher-latitude carbonate formation (cold water carbonates, to which you refer early in the manuscript). Therefore, the uniformitarian approach would be that these high latitude successions are also dominated by cold-water carbonate deposition.

However, to even approach this uniformitarian argument, you need to be able to answer the question (and I have no idea what the answer is): what does a cold-water carbonate looks like in the Precambrian? This is a great question, and one I ask students every time I teach about carbonate rocks. In the absence of the metazoan organisms that define cold-water carbonate facies today? What do you look for? At the minimum, you need to make a facies argument that these high latitude facies are fundamentally different in character than similarly aged low-latitude facies.

(3) Finally, in terms of any of these arguments, you need to address other possibilities. A similar "climate paradox" has been argued in the past about "cap carbonate" facies directly following Neoproterozoic low-latitude glacial (snowball Earth) events. This has mostly gone to the wayside in the literature, in favor of either arguing about the timing and number of such events, or arguing unique geochemical conditions that permitted carbonate formation in the aftermath of global glaciation.

So, in the end. Do I think that there is something special about these high-latitude carbonate deposits – absolutely! Do I think that there is sufficient argument laid out in this manuscript – unfortunately, no.

I would love to read a revised version, however, in the future. You have something here....you just need the right framework for it! Good luck!

Reviewer #2 (Remarks to the Author):

This is a well-written and useful manuscript that lays out a new constraint on Mid-Proterozoic climate. The authors report paleomagnetic data that at least purports to show that typical warm-water carbonate platforms were constructed in India at 1.2 Ga when that land mass was north of 70 degrees latitude. If correct, this shows that the polar oceans at that time were at least as warm as during the Mid-Cretaceous maximum, around 100 Ma. This is a useful target for climate modelers, including this reviewer. There is an ongoing debate about the nature of that climate, which should have depended on greenhouse gas concentrations like CO2, CH4, and N2O, but also on O2 because the latter gas would have influenced the concentrations of the last two of these three greenhouse gases.

I am not going to comment on the observations themselves, as this is best left to other experts in these fields. I hope that the paper is reviewed by a qualified paleomagnetist, as this is a difficult subject that requires expert training. Likewise, the interpretation of what types of carbonate deposits formed at what ocean temperatures can be best addressed by a reviewer with a strong sedimentology/stratigraphy background. Suffice it to say that to a non-expert in these fields, the study seemed carefully done.

The section on the 'Paleoclimate paradox' is also well-written, but I do have two detailed comments that are listed below. The first concerns possible evidence for glaciation during the Boring Billion. The second points to a recent 3-D, Mid-Proterozoic climate calculation by some Chinese researchers. The latter project was led by Peng Liu, who spent two years working with me here at Penn State, which is why I know of this work. It might be useful to include some of these references. This reviewer is particularly interested in whether the evidence for glaciation at 1.8 Ga and 1.1-1.3 Ga should be considered credible.

Finally, I was pleased to see that the manuscript is dedicated to Nic Beukes. I met Nic almost 40 years ago as a member of Bill Schopf's Precambrian Paleobiology Research Group at UCLA. Nic was a valued member of that team, and he continued to provide valuable advice, and to show other Precambrian geologists around South Africa, for many years after that time. We were all sad to learn of his passing. You may reveal my name to the authors.

Jim Kasting
Penn State

Detailed comments:

1. (l. 183) 'The Mesoproterozoic has long been considered a warm time based on the absence of glaciation(2,8).'

--Refs. 2 and 8 are to Holland (2006) and Young (2013). This is indeed the currently accepted story. But in writing our 2017 book, Atmospheric Evolution on Inhabited and Lifeless Worlds', David Catling and I discovered references to papers that question this idea, or at least show some of its limitations (see Sect. 11.3.1, p. 302-303). More detail follows: i) Two of these papers concern possible glaciation in the King Leopold formation in northwestern Australia (G.E. Williams, J. Geol. Soc. 162, 111-124, 2005; Schmidt and Williams, Precambrian Res. 167, 267-280, 2008). This may be sufficiently removed from the middle of the 'Boring Billion' period to not break up the story too much, as the time span of the Boring Billion is often cited as 1.8-0.8 Ga. Are these papers believable? ii) A third paper concerns glaciation in the Vazante group in Brazil, which was previously considered Neoproterozoic in age but has been redated at 1.1-1.3 Ga by N.J. Geboy et al., Precambrian Res. 238, 199-213, 2013). This is right during the time in which the high-latitude carbonates in the present paper are reported. Is this report believable and, if so, does it cast doubt on the conclusions reached in this new manuscript?

2. (Same paragraph) Some additional constraints on Mid-Proterozoic temperatures are provided by 3-D climate simulations by P. Liu, Y. Liu, Y. Hu, J. Yang, S. A. Pisarevsky, Warm climate in the "Boring

Billion" era, Acta Geologica Sinica-English Edition 93, 40–43 (2019). According to their (zero-CH4) climate model results, 3,550 ppmv CO2 at 1.42 Ga would have produced a glacial climate with a mean surface temperature of 10.7 °C, whereas 7,100 ppmv CO2 would have produced a mean surface temperature of 18.6 °C—sufficient to keep the continents (but not the poles) ice free. These calculations account for the fact that solar luminosity at that time was ~90% of the present value. These two CO2 levels are equivalent to 12-24 PAL, if the Preindustrial Atmospheric Level (PAL) is rounded to 300 ppmv. Thus, if these climate calculations are accurate, the new climate constraints provided in the present manuscript would likely require >24 PAL of CO2 and/or additional greenhouse warming by CH4 or N2O.

Reviewer #3 (Remarks to the Author):

Review of "Extreme high-latitude platform carbonate deposition …" by de Kock et al. submitted to Nature Communications

Primarily on the basis data on paleopole positions in late Mesoproterozoic, compiled from the literature and one new set from India, and, the distribution of carbonate platforms of the time, the authors boldly claim that the then poles were not only ice-free but were also warmer than 20°C. On the basis of theoretical arguments, they further infer that the atmosphere of the time was charged with super-greenhouse gases.
Because I have no training in or insight into paleomagnetism, I am accepting the authors' results, but other reviewers need to assess this main part of the paper. As an outside reader, I suggest that the authors consider stating upfront what the error-bars on paleopoles are (fig. 3C) and what their implications might be. It appears that there is an implicit assumption that paleopole positions of a cratonic block determined from a set of dykes must also be those of carbonate deposits in the same block. In the understandable absence of absolute ages of the carbonates (see below), this assumption should also be stated upfront.
The ages and paleopole positions of the igneous rocks are well constrained by using the ~same rock units, for example the Harohalli dykes (zircon; U-Pb), and by using corrections if any. Ages of carbonate platforms discussed in this paper are not as well constrained as the authors rightly state (e.g., lines 49-78). The best age-estimates come from the U-Pb ages of zircons and monazites in the tuffs near the bottom and the top of some basin in the Bastar craton. Detrital zircon ages from siliciclastic units provide maximum depositional ages; they should not be construed to be close to true depositional ages as the authors state in lines 59-61 (the statement must be corrected). Overall however, I agree that carbonate deposition occurred around 1.2 Ga in different cratons in India, and that the authors are within permissible limits in their inferences.
The authors need to correct the spelling of Chhattisgarh.
Incidentally, I suppose that the e-compositor will straighten out many space-obliterations in the text that I received.

Abhijit Basu, Indiana University
October 18, 2023

Reviewer #4 (Remarks to the Author):

The manuscript contains some new paleomagnetic data from Mesoproterozoic Indian carbonates and an interesting observation about their high paleolatitudes. Unfortunately, the age uncertainties of these data are large, so more studies are required to accept the main suggestion of the manuscript about climatic paradox in late Mesoproterozoic. However, it is suitable as a hypothesis and worth publishing after a moderate to major revision.

COMMENTS

Unfortunately the organization of the manuscript is not perfect. It is really hard for readers (and for a reviewer) to read three separate texts in parallel (the main one and two supplementary ones), each with separate illustrations and separate, though overlapping reference lists (some references are present in all three lists, having different numbers in each). In addition there is a table S3 with the fourth reference list. All these parts are important for an understanding of the authors' conception, justification of their ideas and specific details, so it is quite an effort to jump from one part to another all the time. I understand that this problem is partly caused by the strict rules for publications in NC, however there is a challenge for the reader.

Main text

Lines 74-78: the ~1.2 Ga age of the Narji Limestone (NL) is poorly justified. Why not ~1.4 Ga, for example - closer to the mean of 913 (above NL) and 1717 (below NL) Ma detrital zircons? More explanations are needed here.

Lines 80-85: be more specific, what is new in you manuscript compare to previous publication [5].

Lines 103-106: did you re-apply the fold and/or conglomerate tests (according to line 20 in Supporting information: Methods, you collected more conglomerate samples) with inclusion of new data, or just relied on the test results in [5]? If not, I would recommend to do that.

Line 109: add the table of poles (with ages) for Fig. 3C.

Lines 119-120: "…age is otherwise not well constrained" – this is also applicable to C&PG.

Line 122: see comment to line 109. Table of poles is needed here.

Lines 127-128: in view of huge age uncertainties, I would add "at least partly".

Lines 146, 387-396 and Fig. 3C: provide Euler's rotation parameters please.

Line 150: see general comment to "Methods".

Line 157: instead of "within the tropics" I would say more cautiously "at < 50 degrees latitudes".

Line 162: I would say "could also be affected"

Lines 165-166: I propose to rephrase this sentence (it is not only about continents): "The area on the globe on high latitudes is less than area in the low latitudes" – something like this.

Lines 174-176: It would be good to cite Abrajevitch and Van der Voo (EPSL 2010, 293, 164–170). They were the first.

Line 206: …southern India and North China.

Line 236: correct reference is: Geol. Mag. 152(4), 728-750. Also – line 221 (in Supporting Information: Results—Paleomagnetism and rockmagnetism); line 184 (in Supporting Information: Proterozoic carbonate platform paleolatitudes (Bibliography))
Fig. 4 and Table S3: why some vertical error bars are asymmetrical? If we suppose the Fisherian distribution for paleomagnetic directions, dp should be the half of this bar.

Supporting Information: Methods

General comment: please describe how did you calculate the vertical error bars (+ and -) for the paleolatitude. The commonly used method assumes the Fisherian distribution of paleomagnetic directions and consider the minimal axis of the oval of confidence for the pole (dp) as a half of this error bar, as the oval is oriented "perpendicularly" to the arc of great circle connecting the sample location and paleomagnetic pole. However, in this case the vertical "+" and "-" in S3 should be the same and vertical error bars in Fig. 4 should be symmetrical.

Fig. S1 caption: sample localities (*Wabo et al., 2021) - according to reference list(s) Wabo et al., 2022.

Line 85 and Table S3: it would be very useful for readers if Table S3 contain a field with RESULTNO (reference 8) for the data available in the Online GPMDB. Interested readers then will be able to extract much more information about paleomagnetic studies of their interest mentioned in Table S3.

Lines 101-102: some are updated. I propose "… are not always updated…"

Supporting Information: Results—Paleomagnetism and rockmagnetism

Table S1 needs captions. Apparently the superscript [2] in the first column means that these entries are taken from previous study, which is number 2 in the Reference list to this supporting information, but it is number 5 in the Reference list for the main manuscript, and number 59 in the reference list for the supporting Information: Proterozoic carbonate platform paleolatitudes which is not very convenient to the reader/reviewer. In the table caption it would be good to refer to the tables in the study [2], e.g. Table 3 for the high-T component.

Lines 131-135: see my comment to lines 103-106 in the main text.

Lines 152-158, Table S1: apparently only the site KBA (sandstone) represents the clastic sediments in this study, others are carbonates. The KBA inclination is much less than inclinations in other sites (high-T component). Maybe there is something to do here with inclination compaction correction, which was found in some clastic rocks? Maybe if you exclude this site, the reversal test became positive?

Lines 176-191 and Fig. S8(B): It would be much better to add a table (with ages) of used Indian poles in this supporting information.

RESPONSE TO REVIEWER COMMENTS

Response to comments by Reviewer #1:

First, I would like to thank the editors for the opportunity to read this interesting manuscript. I have been overcommitted recently, but I could not turn down a manuscript with such an interesting title.

We thank Reviewer #1 for taking the time to provide a detailed review that raise important criticism of the manuscript.

Second, I would like to commend the authors on an interesting idea. You will see, below, that I do not think this work is ready for publication, but it is an intriguing idea and one that is innovative and has not been argued before.

We are glad that our idea was found to be intriguing and recognized as innovative. In addressing the comments and points raised by the reviewer, we produced a more convincing argument.

Third, I have made extensive comments in the PDF of the manuscript. Please use these line comments during any revisions that you might make.

The individual comments made in the PDF of the manuscript are addressed in detail after replying to three "critical elements" below.

I will only report the **critical elements** that lead to a decision of "not yet publishable" below:

(1) The first critical issue is the assertion that high-latitude carbonate deposition requires globally high temperatures. This is really the crux of the manuscript, but the hypothesis is not well supported (rather, it is simply asserted).

Point 1: The high-latitude carbonate deposition presented in the manuscript requires globally higher temperatures, because the facies is distinct from what can be expected from cold-water carbonate deposits. James and Lukasik (2010) lists characteristics of carbonate deposits in warm temperate, cool temperate and polar regions. We consider those that do not involve Metazoan components as valid also for Precambrian deposits:

- *Interbedded glacial deposits (polar and cool temperate)*
- *Ice-rafted debris (polar)*
- *Minor carbonate mud (polar, cool temperate, and warm temperate)*
- *Local hardgrounds (cool and warm temperate)*
- *Glendonites (polar)*
- *Minor photozoan elements are found in warm temperate settings*

The carbonates described in the manuscript are voluminous, laterally extensive and generally lack reworked clastic carbonate components. They are microbial (with rare spatially limited columnar and branching stromatolite occurrences) and with a high component of carbonate mud. They are free of interbedded glacial deposits and ice-rafted debris. Glendonites have not been documented and neither have local hardgrounds been identified in the facies. As such they have more in common with warm water carbonate facies, but we cannot completely rule out a warm temperate setting either. Given the reported polar paleolatitude, the facies implies a

temperature of at least 15-20°C, if not higher. As such, climate had to be significantly hotter than today, especially if the effect of a ~10% lower solar luminosity is considered.

[James, N. P. and Lukasik, J., 2010. Cool- and cold-water neritic carbonates, in James, N. P. and Dalrymple, R. W., eds., Facies Models 4. Geological Association of Canada, 271-399.]

The absence of glacial activity during this time does not require dramatically higher temperatures. Polar glaciation also requires the existence of sufficient continental mass as reservoirs for ice (i.e. like Antarctica), or sufficient continental mass that restricts active flow of oceanic currents to higher latitudes (i.e. like the northern polar ocean). At times where there is little polar continental mass, oceans tend to not form ice, and are generally warmer, but need not be drastically so.

The Mesoproterozoic was for the greater part free of glaciation, and is generally regarded in literature as a warmer time. It is, however, true that the absence of glacial activity does not require dramatically higher temperatures, but it is important to be cognizant of the effects of ~10% lower solar luminosity during this time.

Unfortunately, continental reconstructions at the moment are not able to provide enough granularity to allow for conclusive statements about ocean currents, but in terms of continental mass at the poles, we illustrate that southern cratonic India as well as North China likely occupied polar latitude in the same hemisphere at the end of the Mesoproterozoic. This would have increased the likelihood of glaciation, yet no evidence for this has been forthcoming.

Climate modelling by Liu et al. (2019) take lower solar luminosity into account and has shown that pCO_2 at 12 times PAL would produce a glacial climate. Ice-free continents (but not if they were situated at the poles) would have required 24 times PAL. Ice-free southern India and North China near the pole thus would have required more than 24 times PAL of pCO_2. We therefore think that an ice-free end-Mesoproterozoic probably required significantly higher temperatures. The carbonate facies of the Purana platform does not contradict this view.

[Lui, P., Lui, Y., Hu, Y., Yang, J. and Pisarevsky, S., 2019. Warm Climate in the "Boring Billion" Era. Acta Geologica Sinica, 93, 40-43.]

(2) The second critical issue is the assertion that warmer temperatures (see above) is the uniformitarian approach. In fact, it is not. On the modern Earth, we have higher-latitude carbonate formation (cold water carbonates, to which you refer early in the manuscript). Therefore, the uniformitarian approach would be that these high latitude successions are also dominated by cold-water carbonate deposition.

However, to even approach this uniformitarian argument, you need to be able to answer the question (and I have no idea what the answer is): what does a cold-water carbonate looks like in the Precambrian? This is a great question, and one I ask students every time I teach about carbonate rocks. In the absence of the metazoan organisms that define cold-water carbonate facies today?
What do you look for? At the minimum, you need to make a facies argument that these high latitude facies are fundamentally different in character than similarly aged low-latitude facies.

Point 2: The reviewer is correct that a uniformitarian approach, in the strict sense, would be to regard the high-latitude carbonate succession of the Purana basins as cold-water carbonate deposits. We now acknowledge this view explicitly in the revised manuscript. However, the Purana carbonate facies is distinct from the facies expected for Precambrian cold-water deposition (see the reply at point 1, above). The Purana facies is analogous to modern warm-water carbonate facies, but we also cannot conclusively rule out a warm temperate setting. We thus apply a uniformitarian approach in the loose sense of inferring modern conditions (specifically, water ocean temperature) to the observed ancient facies.

(3) Finally, in terms of any of these arguments, you need to address other possibilities. A similar "climate paradox" has been argued in the past about "cap carbonate" facies directly following Neoproterozoic low-latitude glacial (snowball Earth) events. This has mostly gone to the wayside in the literature, in favor of either arguing about the timing and number of such events, or arguing unique geochemical conditions that permitted carbonate formation in the aftermath of global glaciation.

Point 3: Given the current poor documentation of cold-water Precambrian carbonate facies, we acknowledge that there is some uncertainty to our interpretation of Purana carbonate deposit. However, based on the facies argument as outlined above in points 1 and 2, we consider polar ocean water temperatures of at least above 15°C, but likely higher than 20°C a prerequisite. As such the deposits are a proxy for greenhouse climatic conditions that required higher pCO2 (and other greenhouse gasses) than previously considered for the Mesoproterozoic.

We further acknowledge that, an explanation for our high-latitude carbonate deposits need to go beyond ocean temperature. Other potential controls like carbonate saturation, ocean circulation or continental configuration should be considered in the future. These factors require a global perspective and level of detail that is not provided just yet by current continental reconstructions.

We also do list several alternative non-actualistic possibilities, and explain why we regard them as unlikely explanations for the late Mesoproterozoic carbonate deposits of southern India and North China.

Given the implications of using the word “paradox” we have opted for a softer expression and replaced it with “conundrum” in all instances.

So, in the end. Do I think that there is something special about these high-latitude carbonate deposits – absolutely! Do I think that there is sufficient argument laid out in this manuscript – unfortunately, no.

I would love to read a revised version, however, in the future. You have something here....you just need the right framework for it! Good luck!

We are excited to note that the significance of the high-latitude carbonate deposits described in our manuscript is obvious to the reviewer, and would like to thank him/her once again for providing us with the guidance to present a better argument and framework for our preferred interpretation.

Detailed comments:

Line 13: There is no sense in using the phrase "boring billion" when: (1) it is clearly used out of context, and (2) it is clearly not true. Calling the Mesoproterozoic the "Dullest time in Earth History" (Buick et al 1995) was in reference to the carbon isotope records of the late Paleoproterozoic and early Mesoproterozoic (and shown not to extend into the latter Mesoproterozoic by Kah et al. in 1999). The phrase "boring Billion" was coined by Bob Hazen, to describe a time interval when the inventory of new minerals was at a minimum. If you are talking about the Mesoproterozoic here, just use Mesoproterozoic.

Although the term "boring billion" is still very much in use today, we agree with the reviewer that this time interval was anything but boring. We also agree that it is not completely necessary to use the term in this manuscript. We have changed it to "Mesoproterozoic".

Line 15: *"Substantial" instead of "Significant"*

Line 18: "Previous studies[4-6] noted ~~such~~ high latitudes"

Done.

Line 22: "high-latitude carbonate~~s~~ represent a paleoclimate paradox"

Done.

Line 22: "high-latitude carbonate~~s~~ represent a paleoclimate paradox"
This is going to need explained. Why is this paradoxical? Because carbonate deposits are typically tropical (not necessarily -- cf. cold-water carbonate deposits of James et al.)

See our reply to the critical elements in points 1-3, above. We discuss cold-water carbonate deposits in some detail, and have highlighted why we think our carbonate facies is more likely to be from warm-temperate to warm water settings. Given the implications of using the word "paradox" we have opted for a softer expression and replaced it with "conundrum" in all instances.

Line 23: Carbonate rocks form today in many environments (deep ocean, higher latitudes) where ocean temperatures are certainly less than 20°C. Carbonate deposits also formed in the aftermath of snowball earth events (also often called a climate paradox), but to make these a paradox, you need to believe that there is not another mechanism that could account for their deposition.

See our reply to the critical elements in points 1-3, above. Here we explain why we regard the deposits as out of the ordinary. If our interpretation is correct, they are "paradoxical" in that they require higher atmospheric pCO_2 levels than what is currently excepted for the late Mesoproterozoic. Given the implications of using the word "paradox" we have opted for a softer expression and replaced it with "conundrum" in all instances.

Line 31: Better to say "biotic constituents" because some of the "abiotic" constituents (e.g. carbonate mud) are found in high latitudes (cf. O'Connell et al), and extent of cementation (which is often said to vary) has not been well-described.

We implemented the change of wording to "biotic constituents" as opposed to just "constituents".

The identification of carbonate mud in high-latitude settings by O'Connell et al. (2015) is noted and now referred to in the revised manuscript. It is, however, noted that the mud-sized carbonate particles are the product of skeletal fragment breakdown.

The question I always ask is....how would one recognize a cold-water carbonate in the Precambrian?

See our reply to the critical elements, point 1.

Line 32: *"comprised largely of" instead of "essentially"*

Line 32: *"bioclasts" instead of "bioclastics"*

Line 32: "ahermatypic coral~~s~~"

Done.

Line 33: As noted above, this is not necessarily true. [regarding lime-mud]

We added the statement: "Carbonate mud, which is usually a minor component of high-latitude Phanerozoic carbonate deposits has been interpreted as the product of skeletal fragment breakdown."

Line 35: "~~photosymbiont~~"

Done.

Line 36: "hermatypic coral~~s~~"

Done.

Lines 37-39: I know you are making an argument, but this ignores decades of work on cool-water carbonate facies.

The statement that warm-water neritic environments are the principal settings for carbonate production and preservation is based on the analysis by Kiessling et al. (2003). Perhaps it is better to say that warm-water neritic environments are responsible for most carbonate production and preservation in terms of volume.

Work done on cold and cool-water carbonate facies are discussed in some detail.

[Kiessling, W., Flügel, E. and Golonka, J. A. N., 2003. Patterns of Phanerozoic carbonate platform sedimentation. Lethaia, 36, 195-225.]

Lines 39: "This is also assumed" Please be discrete -- what is assumed? Tropical carboante deposition?

We now make it clear that it is the preferential tropical deposition of Precambrian platforms that is assumed by paleogeographers. We note that this may be a false assumption as cold water carbonate facies from the Precambrian are not well documented.

Line 42-43: Regarding aragonite fans: Also Mesoproterozoic and Neoproterozoic -- could cite recent compilations like Cantine et al. (2019) and Kah and Bartley (2021).

In an attempt to limit the number of citations (given the constraints imposed by Nature Communications) we rather opted to say that aragonite fans formed during the Precambrian. As such we are inclusive of the Archean as well as the whole of the Proterozoic Era.

Line 43: What is "they" here?

In the revision we make it clear that we are referring to Precambrian carbonate platforms.

Line 45: *“pattens” was replaced with “patterns”*

Line 50: *Comma added after India*

Line 54: *“A succession of” was added before “carbonate and shale”*

Line 54: *“facies” instead of “carbonates”*

Line 60: *Comma added before and after “too”*

Line 62: What is "it" here? The Purana platform cover in the Kaladgi, Bhima, and Cuddapah basins?

The statement was revised to clarify that the timing of the platform’s deposition in the Kaladgi, Bhima and Cuddapah basins is the subject here.

Line 70-73: Just thinking ahead. At present, this is not enough of a description to place this into any sort of platform context.

We agree that the description on its own is not enough to place the units within a platform context, but this has been done by the detailed sedimentological work of previous studies on which we rely. It is also only after the Kurnool Group is considered in the context of its correlation to the other Purana basin successions that the large lateral extent and voluminous nature of the platform becomes apparent. In addition, further description and details are provided in Figure 2 and its caption. These carbonates were mostly deposited below wave base and are bedded lime mudstones with spatially limited columnar and branching stromatolite occurrences, calcareous shales, and locally developed lime-clast debris-flow conglomerates and calciturbidites in distally steepened settings. The facies is interpreted as being deposited in a carbonate ramp environment.

In order to avoid confusion regarding terminology we provide the following statement in the introduction: “Several terms exist for neritic carbonate deposits based on geometry (i.e., ramps, rimmed and open shelfs), but we generically use the term “platform”.

Line 76: *“minimum” was replaced with “maximum”*

Line 85-88: *We are pleased that the reviewer likes our compilation of Precambrian carbonate platform paleolatitudes.*

Line 97-98: *The steep primary magnetizations were indeed surprising.*

Line 106-109: How different? Is there evidence of a very large shift in pole location between 1400 and 1075?

A large shift in pole location is what is observed between 1400 and 1075 Ma (Fig. S.8B). At both these instances southern India was located at a lower or equatorial latitude, respectively.

Does the character of carbonate change during this time?

There are no carbonate units in the Purana succession that are ~1.4 Ga in age. Limestone deposited near the upper part of Kurnool Group is likely around ~1.0 Ga, but not constrained by any paleomagnetic data. The carbonate facies here and in the other Purana basins (i.e., upper parts of the Raipur Group, Indravati Group and the Pranhita-Godavari Valley Basin) are dominated by pink-reddish calcareous shale.

Line 120-124: OK, but you previously said that the pole was very different in character from Indian poles at 1465, 1075, and 770.

The implication is that the age of magnetization and the Purana platform is likely removed from the current maximum (1405 ± 9 Ma) and minimum (1001 ± 7 Ma) age constraints, but closer to 1.2 Ga as also indicated by the ~1180 Ma glauconite age from the Pranhita-Godavari Valley. We thus tentatively assign the 1192 ± 10 Ma age of the Harohalli alkaline dyke pole to our pole. This age is, however, subject to debate and we remain cognizant of the wider age range when considering the Purana platform's paleomagnetic constraints.

Line 126: Is this your work, or is there a citation missing here?

We have added references for the paleolatitude of the Chhattisgarh and Pranhita-Godavari Valley basins and the Kaladgi and Bhima basins.

Line 126: *"carbonates" was changed to "carbonate facies"*

Line 143-146: OK. This is not a lot to go on for the interpretation, but North China has always been a bit of a problem.

A change from purplish-pink to grey limestone is seen in both the Purana platform as well as the North China platform. This, together with the paleomagnetic correlation and near coevality of the two successions is suggestive of a shared history.

Line 147: If this is plural, please cite more than one paper.

We revised the text to now read: "Such a near-neighbour relationship has been noted before, albeit in a different configuration"

Line 157-160: Whereas this is true, the more pressing issue is our ability to determine what a cold-water carbonate would look like in the Precambrian....this has not really been discussed at all, yet.

See our reply to critical elements, point 1. Cold-water carbonates and what they might look like in the Precambrian is now discussed in the revision.

Line 161-163: Whereas this is true, these are broadly epicontinental successions within rift basins, so this argument does not really apply.

This was also commented on by Reviewer #4. We have revised and shortened the text to read: "deposition … could also be affected by the supercontinent cycle".

Line 167-168: OK, so I am mostly through the manuscript before you really come down and tell your audience what is going on....and it is this: You assert that these high-latitude carbonate platforms indicate a much hotter earth.

This "punchline" is now stated already in the abstract, while also mentioning other possible interpretations. In the discussion we clarify why we think these facies are not representative of the cold-water realm, but rather a warm-temperate to warm-water realm. See our reply to critical elements.

Unfortunately, you have not given the reader any argument for this possibility. Only an unsupported assertion.

We have now articulated our argument more clearly and support it with a facies argument.

Lines 156-169: Much of these arguments are pro-forma and not very interesting. Could be condensed.

The section was condensed.

Figure 3C: OK, I am a bit behind on new reconstructions from Paleomag, but this is even a very different reconstruction than that given in the Chinese work that is cited here for the high latitudes of the North China platform.

In the text we note that "we reconstruct India and North China as near neighbours during the assembly of Rodinia. Such a near-neighbour relationship has been noted before, albeit in a different configuration" to that proposed by Zhao et al. (2018).

Paleomagnetic reconstruction of continental blocks are non-unique with many degrees of freedom, particularly if continental blocks are restored at a specific time (i.e., closest approach reconstruction method) as opposed to over a longer time interval (i.e., apparent polar wander path matching reconstruction method).

Our reconstruction of North China relative to the better constrained core of Rodinia (i.e., Laurentia, Siberia, and Baltica) follows Zhang et al. (2021) and is also illustrated by Evans (2021):

Zhang et al. (2021)

REDACTED

Evans (2021):

REDACTED

[Zhang, S., Chang, L., Zhao, H., Ding, J., Xian, H., Li, H., Wu, H. and Yang, T., 2021. The Precambrian drift history and paleogeography of the Chinese cratons, in Pesonen, L. J., Salminen, J., Elming, S. Å., Evans, D. A. D. and Veikkolainen, T., eds., Ancient Supercontinents and the Paleogeography of Earth. Elsevier, 333-376.]

[Evans, D. A. D., 2021. Meso-Neoproterozoic Rodinia supercycle, in Pesonen, L. J., Salminen, J., Elming, S. Å., Evans, D. A. D. and Veikkolainen, T., eds., Ancient Supercontinents and the Paleogeography of Earth. Elsevier, 549-576.]

Response to comments by Reviewer #2:

This is a well-written and useful manuscript that lays out a new constraint on Mid-Proterozoic climate. The authors report paleomagnetic data that at least purports to show that typical warm-water carbonate platforms were constructed in India at 1.2 Ga when that land mass was north of 70 degrees latitude. If correct, this shows that the polar oceans at that time were at least as warm as during the Mid-Cretaceous maximum, around 100 Ma. This is a useful target for climate modelers, including this reviewer. There is an ongoing debate about the nature of that climate, which should have depended on greenhouse gas concentrations like CO_2, CH_4, and N_2O, but also on O_2 because the latter gas would have influenced the concentrations of the last two of these three greenhouse gases.

I am not going to comment on the observations themselves, as this is best left to other experts in these fields. I hope that the paper is reviewed by a qualified paleomagnetist, as this is a difficult subject that requires expert training. Likewise, the interpretation of what types of carbonate deposits formed at what ocean temperatures can be best addressed by a reviewer with a strong sedimentology/stratigraphy background. Suffice it to say that to a non-expert in these fields, the study seemed carefully done.

Reviewer #1 commented on the carbonate sedimentology, while Reviewer #3 commented on the stratigraphy. Reviewer #4 commented on aspects of the paleomagnetism.

The section on the ‘Paleoclimate paradox’ is also well-written, but I do have two detailed comments that are listed below. The first concerns possible evidence for glaciation during the Boring Billion. The second points to a recent 3-D, Mid-Proterozoic climate calculation by some Chinese researchers. The latter project was led by Peng Liu, who spent two years working with me here at Penn State, which is why I know of this work. It might be useful to include some of these references. This reviewer is particularly interested in whether the evidence for glaciation at 1.8 Ga and 1.1-1.3 Ga should be considered credible.

See responses to the detailed comments below.

Finally, I was pleased to see that the manuscript is dedicated to Nic Beukes. I met Nic almost 40 years ago as a member of Bill Schopf’s Precambrian Paleobiology Research Group at UCLA. Nic was a valued member of that team, and he continued to provide valuable advice, and to show other Precambrian geologists around South Africa, for many years after that time. We were all sad to learn of his passing. You may reveal my name to the authors.

Jim Kasting
Penn State

We thank Prof Kasting for his positive review of the manuscript and for his constructive detailed comments below. We addressed these in our revision.

<u>Detailed comments:</u>

1. (l. 183) ‘The Mesoproterozoic has long been considered a warm time based on the absence of glaciation(2,8).’

--Refs. 2 and 8 are to Holland (2006) and Young (2013). This is indeed the currently accepted story. But in writing our 2017 book, Atmospheric Evolution on Inhabited and Lifeless Worlds', David Catling and I discovered references to papers that question this idea, or at least show some of its limitations (see Sect. 11.3.1, p. 302-303). More detail follows: i) Two of these papers concern possible glaciation in the King Leopold formation in northwestern Australia (G.E. Williams, J. Geol. Soc. 162, 111-124, 2005; Schmidt and Williams, Precambrian Res. 167, 267-280, 2008). This may be sufficiently removed from the middle of the 'Boring Billion' period to not break up the story too much, as the time span of the Boring Billion is often cited as 1.8-0.8 Ga. Are these papers believable? ii) A third paper concerns glaciation in the Vazante group in Brazil, which was previously considered Neoproterozoic in age but has been redated at 1.1-1.3 Ga by N.J. Geboy et al., Precambrian Res. 238, 199-213, 2013). This is right during the time in which the high-latitude carbonates in the present paper are reported. Is this report believable and, if so, does it cast doubt on the conclusions reached in this new manuscript?

We note the reviewer's concern regarding the statement that the Mesoproterozoic is considered warm based on the absence of glaciation. The statement was revised to read: "The Mesoproterozoic has long been considered a warm time based on the general absence of glaciation at a time of lower solar luminosity". This statement now accommodates the isolated reports of possible glaciation mentioned by the reviewer. The 1.0-1.1 Ga possible glacial unit occurrences are also now listed in the revised manuscript. The 1.8 Ga King Leopold glaciation is far removed from the time interval that is the focus of this manuscript but is indicated on Figure 4. The purported 1.0-1.1 Ga Vazante Group glacial record is complicated in that the São Fransisco craton was probably located at low latitude at the time in question (See figure below from Trindade et al., 2021) and adds to the climatic conundrum.

REDACTED

[Trindade, R. I. F., D'Agrella-Filho, M. S., Antonio, P. Y. J. and Teixeira, W., 2021. The Precambrian drift history and paleogeography of Congo-São Francisco craton, in Pesonen, L. J., Salminen, J., Elming, S. Å., Evans, D. A. D. and Veikkolainen, T., eds., Ancient Supercontinents and the Paleogeography of Earth. Elsevier. Amsterdam, Netherlands, 445-464.]

2. (Same paragraph) Some additional constraints on Mid-Proterozoic temperatures are provided by 3-D climate simulations by P. Liu, Y. Liu, Y. Hu, J. Yang, S. A. Pisarevsky, Warm climate in the "Boring Billion" era, Acta Geologica Sinica-English Edition 93, 40–43 (2019). According to their (zero-CH4) climate model results, 3,550 ppmv CO_2 at 1.42 Ga would have produced a glacial climate with a mean surface temperature of 10.7 °C, whereas 7,100 ppmv CO_2 would have produced a mean surface temperature of 18.6 °C—sufficient to

keep the continents (but not the poles) ice free. These calculations account for the fact that solar luminosity at that time was ~90% of the present value. These two CO2 levels are equivalent to 12-24 PAL, if the Preindustrial Atmospheric Level (PAL) is rounded to 300 ppmv. Thus, if these climate calculations are accurate, the new climate constraints provided in the present manuscript would likely require >24 PAL of CO2 and/or additional greenhouse warming by CH4 or N2O.

We thank the reviewer for bringing our attention to the interesting climate model of Liu et al. (2019). The results of these simulations have now been worked into the revised manuscript.

Reviewer #3 (Remarks to the Author):

Review of "Extreme high-latitude platform carbonate deposition …" by de Kock et al. submitted to Nature Communications

Primarily on the basis data on paleopole positions in late Mesoproterozoic, compiled from the literature and one new set from India, and, the distribution of carbonate platforms of the time, the authors boldly claim that the then poles were not only ice-free but were also warmer than 20°C. On the basis of theoretical arguments, they further infer that the atmosphere of the time was charged with super-greenhouse gases.

We would like to thank Reviewer #3 for reviewing the manuscript and for the valuable constructive criticism. We have addressed all the issues raised.

It is noted that we follow previous studies that claim generally ice-free conditions during much of the Mesoproterozoic. Our illustration of the occurrence of laterally extensive and thick carbonate deposition at very high latitude without documented glendonite occurrences or interbedded glacial sedimentary units supports the idea that at least one of the poles was unglaciated and that indeed the Earth was probably experiencing greenhouse climatic conditions. It is further noted that we revised the use of the wording "super greenhouse" to simply "greenhouse" in our revision as it has been brought to our attention that "super greenhouse" has a very specific meaning in climate science. "Super greenhouse conditions" imply a positive feedback of greenhouse warming due to connections between increases in temperature and water vapor. This is not the meaning that we intend to convey.

Because I have no training in or insight into paleomagnetism, I am accepting the authors' results, but other reviewers need to assess this main part of the paper.

Reviewer #4 commented on aspects of the paleomagnetism.

As an outside reader, I suggest that the authors consider stating upfront what the error-bars on paleopoles are (fig. 3C) and what their implications might be.

Although we are not completely sure what the reviewer's expectation is here, we do explain in the text that the poles imply a paleolatitude of 72° ±12° for the Kurnool Group deposition, which is comparable to the 72° ±13° and 83° ±16° obtained from the Chhattisgarh and Pranhita-Godavari Valley basins and the Kaladgi and Bhima basins. The implications of this are discussed in the manuscript.

It appears that there is an implicit assumption that paleopole positions of a cratonic block determined from a set of dykes must also be those of carbonate deposits in the same block. In the understandable absence of absolute ages of the carbonates (see below), this assumption should also be stated upfront.

We have added two sentences at the end of the section concerning the age of the pole for the carbonates in the light of the comparison with known paleopoles. The comparison implies that the age of magnetization and the Purana platform is likely removed from the current maximum (1405 ± 9 Ma) and minimum (1001 ± 7 Ma) age constraints, but closer to 1.2 Ga as also indicated by the ~1180 Ma glauconite age from the Pranhita-Godavari Valley. We thus tentatively assign the 1192 ± 10 Ma age of the Harohalli alkaline dyke pole to our pole.

This age is, however, subject to debate and we remain cognizant of the possibe wider age range when considering the Purana platform's paleomagnetic constraints.

The ages and paleopole positions of the igneous rocks are well constrained by using the ~same rock units, for example the Harohalli dykes (zircon; U-Pb), and by using corrections if any. Ages of carbonate platforms discussed in this paper are not as well constrained as the authors rightly state (e.g., lines 49-78). The best age-estimates come from the U-Pb ages of zircons and monazites in the tuffs near the bottom and the top of some basin in the Bastar craton.

This is clearly articulated in the text: "The minimum age limit of the platform is directly constrained by U-Pb zircon crystallization ages from tuffaceous units in the Chhattisgarh and Indravati basins to predate 993 ± 8 million year (Ma) and 1001 ± 7 Ma repectively, and to post-date 1405 ± 9 Ma in the Chhattisgarh Basin. The timing of the platform's deposition is less certain in the Kaladgi, Bhima and Cuddapah basins, but is broadly assigned to the latest Mesoproterozoic to early Neoproterozoic …".

Detrital zircon ages from siliciclastic units provide maximum depositional ages; they should not be construed to be close to true depositional ages as the authors state in lines 59-61 (the statement must be corrected).

This comment applies to constraints on the age of the Kolhan Group The statement concerning detrital zircon ages for the Kolhan Group was revised.

Overall however, I agree that carbonate deposition occurred around 1.2 Ga in different cratons in India, and that the authors are within permissible limits in their inferences.

We are happy to note the reviewer's agreement that the carbonate deposition occurred around 1.2 Ga.

The authors need to correct the spelling of Chhattisgarh.

The spelling of Chhattisgarh was corrected in all instances.

Incidentally, I suppose that the e-compositor will straighten out many space-obliterations in the text that I received.

This is unfortunate. It is noted that space-obliterations were not visible in the approved pdf of the initial submission. This issue was not commented on by the other three reviewers. Perhaps it is due to a display issue or software version related.

Detailed comments:

Line 55: *"upper" was replaced with "minimum"*

Line 82: What are the rock types?

Core plug rock types are now listed.

Line 100: I am not familiar with the conventions in sedimentary paleomagnetic studies. Hence I ask for my education - Are the hematite and magnetite crystals co-precipitates with the carbonates, or, diagenetic, or detrital?

The positive intraformational conglomerate tests indicate that the magnetization carried by hematite and magnetite was recorded at the time of carbonate deposition. The exact nature of the magnetic minerals (i.e, co-precipitates vs. detrital, or even very early diagenetic) is not constrained.

And, are "intraformational conglomerate tests" universally accepted?

The conglomerate test is widely used in palaeomagnetism to date components of natural remanent magnetization with respect to deposition of conglomerates. A positive conglomerate test from an intraformational conglomerate provides very strong evidence for a primary magnetization (Butler, 1992).

[Butler, R. F., 1992. Paleomagnetism: magnetic domains to geologic terranes. Blackwell Scientific Publishers, Boston, 319pp.]

Lines 127-128: It would be fair if the authors draw attention to the uncertainties in the paleopole positions. Most can be considered "high latitude" even at extremes of the "error circles".

We replaced "extreme high latitude" with "high latitude"

Line 133: This is the crux. While Kiessling et al. (2003) summarize the data on Phanerozoic carbonate platforms, acceptance of >50 deg deposits require robust proof. Only reviewers with expertise in paleomagnetism can assess the data; not me.

Reviewer #4 accessed the paleomagnetic data in detail, and accepts that the units were deposited at more than 50° from the equator.

Line 180: Sounds reasonable. GW's hypothesis has not caught on. DADE prevails.

We note the reviewer's general agreement with the statement that high-obliquity and non-axial dipole geomagnetic fields are unlikely during the late Mesoproterozoic.

Line 188: This is a good argument. Regardless of their latitudinal positions, late Mesoproterozoic carbonates are abundant. Atmospheric and sea water conditions must have conspired to aid such deposition.

We note the reviewer's agreement with the argument.

Lines 209-210 Suggest "likely higher than" … at authors' discretion.

Done.

Reviewer #4 (Remarks to the Author):

The manuscript contains some new paleomagnetic data from Mesoproterozoic Indian carbonates and an interesting observation about their high paleolatitudes. Unfortunately, the age uncertainties of these data are large, so more studies are required to accept the main suggestion of the manuscript about climatic paradox in late Mesoproterozoic. However, it is suitable as a hypothesis and worth publishing after a moderate to major revision.

We thank the reviewer for his detailed report on the manuscript and for the valuable constructive comments. The age uncertainties are indeed large and the limitations of this is clearly articulated in the manuscript. We agree with the reviewer that more studies are needed. We hope that acceptance of our manuscript will result in more attention being given to these interesting units from southern India. We, however, do not agree with the reviewer that the climatic implications of high-latitude non-cold-water carbonate deposition are necessarily negatively affected by the current age constraints. These implications remain no matter what the absolute age of the carbonate deposition is within the possible age range between 1.4 and 1.0 Ga. We are therefore pleased to note that the reviewer considers our hypothesis worth publishing.

COMMENTS

Unfortunately the organization of the manuscript is not perfect. It is really hard for readers (and for a reviewer) to read three separate texts in parallel (the main one and two supplementary ones), each with separate illustrations and separate, though overlapping reference lists (some references are present in all three lists, having different numbers in each). In addition there is a table S3 with the fourth reference list. All these parts are important for an understanding of the authors' conception, justification of their ideas and specific details, so it is quite an effort to jump from one part to another all the time. I understand that this problem is partly caused by the strict rules for publications in NC, however there is a challenge for the reader.

The organization of the manuscript is indeed largely dictated by the strict rules for publication in Nature from which the manuscript was transferred. The Supplementary Information was now reorganized into a single PDF document as per formatting instructions of Nature Communications and many of the issues raised by Reviewer #4 are resolved through this. We do, however, believe that the dataset, its interpretation, and discussion can be followed by the general reader without issue (e.g., reviewer 1-3 did not find issues with the organization). The supplementary information is mostly of interest to paleomagnetic specialists, and should not pose a challenge to the wider readership.

Main text

Lines 74-78: the ~1.2 Ga age of the Narji Limestone (NL) is poorly justified. Why not ~1.4 Ga, for example - closer to the mean of 913 (above NL) and 1717 (below NL) Ma detrital zircons? More explanations are needed here.

The interpretation of the ages mentioned by the reviewer are not correct. The 913 Ma and 1717 Ma ages are <u>both</u> ages for detrital zircon grains from the Paniam Formation that is developed <u>above the Narji Formation</u>. The Paniam Formation yielded a single 913 ± 11 Ma zircon grain, but the next youngest age population from several grains at 1717 ± 20 Ma is a

more conservative maximum depositional age estimate for the Paniam Formation. The age constraints thus at best suggest that the Narji Formation is older than 913 Ma.
Our ~1.2 Ga age assignment of the Narji Limestone is justified by means of a comparison to well-established Precambrian paleomagnetic poles from India. The implication of this comparison is that the age of magnetization and the Purana platform (and thus the Narji Limestone) is likely well removed from the current maximum (1405 ± 9 Ma) and minimum (1001 ± 7 Ma) age constraints, but closer to 1.2 Ga. This is based on its pole similarity to that of the Chhattisgarh and Pranhita-Godavari Valley basins as well as the 1192 ± 10 Ma Harohalli alkaline dykes pole. The latter age is now tentatively assigned to the poles of the Purana platform, while remaining cognizant of the possible wider age range. This is now explicitly stated in the text.

Lines 80-85: be more specific, what is new in you manuscript compare to previous publication [5].

It is now made clear that our data is based on more extensive sampling of the Kurnool Group compared to previous work [5]. Our sampling includes new sites and wider stratigraphic coverage of the Kurnool Basin and, importantly, also include results from strata of the Palnad Basin, which was not sampled before. In addition, we present the first rock-magnetic results for the Kurnool Group. This is clearly stated in the revised text.

Lines 103-106: did you re-apply the fold and/or conglomerate tests (according to line 20 in Supporting information: Methods, you collected more conglomerate samples) with inclusion of new data, or just relied on the test results in [5]? If not, I would recommend to do that.

We did not re-apply the fold and conglomerate tests, but did indeed rely on the results of these tests in [5]. These tests are considered robust. Our new data also cannot be directly applied to these tests for a re-evaluation. The reason for this is that the fold structure used for the fold test [5] was a small scale and localized anticlinal structure. The test was conducted at the site level using sample data. Regionally the strata display minimal deformation with dips of less than 10°.

Line 109: add the table of poles (with ages) for Fig. 3C.

A table of poles used in comparison in Fig. 3C is now provided in the supplementary information (Table S2).

Lines 119-120: "…age is otherwise not well constrained" – this is also applicable to C&PG.

We have changed the wording to: "but its age of magnetization is not constrained by field tests". The intention here is to highlight the difference between the K&B basins and the Kurnool Group and the C&PG basins, where the age of magnetizations for the Kurnool Group and the C&PG basins are illustrated to be primary based on intraformational conglomerate tests and a fold test.

Line 122: see comment to line 109. Table of poles is needed here.

A table of poles used in comparison in Fig. 3C is now provided in the supplementary information (Table S2).

Lines 127-128: in view of huge age uncertainties, I would add “at least partly”.

Done.

Lines 146, 387-396 and Fig. 3C: provide Euler’s rotation parameters please.

Euler rotation parameters are provided in the caption.

Line 150: see general comment to “Methods”.

See responses below.

Line 157: instead of “within the tropics” I would say more cautiously “at < 50 degrees latitudes”.

Done.

Line 162: I would say “could also be affected”

Done.

Lines 165-166: I propose to rephrase this sentence (it is not only about continents): “The area on the globe on high latitudes is less than area in the low latitudes” – something like this.

The paragraph was shortened based on the comments of Reviewer 1. The statement now reads: “Other biasing factors are the presence of continents near the poles and the area on the globe at high latitude compared to low latitude.”

Lines 174-176: It would be good to cite Abrajevitch and Van der Voo (EPSL 2010, 293, 164–170). They were the first.

Done.

Line 206: …southern India and North China.

Done.

Line 236: correct reference is: Geol. Mag. 152(4), 728-750. Also – line 221 (in Supporting Information: Results—Paleomagnetism and rockmagnetism); line 184 (in Supporting Information: Proterozoic carbonate platform paleolatitudes (Bibliography))

The reference was corrected in all instances.

Fig. 4 and Table S3: why some vertical error bars are asymmetrical? If we suppose the Fisherian distribution for paleomagnetic directions, dp should be the half of this bar.

We thank the reviewer for highlighting the issue with the vertical error bars. The vertical error bars should be symmetrical and not asymmetrical. They are now represented as ±dp (the minimal axis of the oval of confidence for the virtual geomagnetic pole) or as ±A_{95} (the

radius of the circle of confidence for the paleomagnetic pole). The text, Figure 4, and Table S3 have been corrected accordingly.

Supporting Information: Methods

General comment: please describe how did you calculate the vertical error bars (+ and -) for the paleolatitude. The commonly used method assumes the Fisherian distribution of paleomagnetic directions and consider the minimal axis of the oval of confidence for the pole (dp) as a half of this error bar, as the oval is oriented "perpendicularly" to the arc of great circle connecting the sample location and paleomagnetic pole. However, in this case the vertical "+" and "-" in S3 should be the same and vertical error bars in Fig. 4 should be symmetrical.

We thank the reviewer for highlighting the issue with the vertical error bars. The vertical error bars should be symmetrical and not asymmetrical. They are now represented as ±dp (the minimal axis of the oval of confidence for the pole) or as $\pm A_{95}$ (the radius of the circle of confidence for the paleomagnetic pole). The text, Figure 4, and Table S3 have been corrected accordingly.

Fig. S1 caption: sample localities (*Wabo et al., 2021) - according to reference list(s) Wabo et al., 2022.

The reference in the figure legend was corrected to Wabo et al., 2022.

Line 85 and Table S3: it would be very useful for readers if Table S3 contain a field with RESULTNO (reference 8) for the data available in the Online GPMDB. Interested readers then will be able to extract much more information about paleomagnetic studies of their interest mentioned in Table S3.

We thank the reviewer for this suggestion. We agree that inclusion of the Online GPMD result numbers would indeed be useful. Table S3 was updated to include a column listing GPMD result numbers.

Lines 101-102: some are updated. I propose "… are not always updated…"

Done.

Supporting Information: Results—Paleomagnetism and rockmagnetism

Table S1 needs captions. Apparently the superscript [2] in the first column means that these entries are taken from previous study, which is number 2 in the Reference list to this supporting information, but it is number 5 in the Reference list for the main manuscript, and number 59 in the reference list for the supporting Information: Proterozoic carbonate platform paleolatitudes which is not very convenient to the reader/reviewer. In the table caption it would be good to refer to the tables in the study [2], e.g. Table 3 for the high-T component.

Captions have been added to Table S1.

Lines 131-135: see my comment to lines 103-106 in the main text.

We did not re-apply the fold and conglomerate tests, but did indeed rely on the results of these tests in [5]. These tests are considered robust. Our new data also cannot be directly applied to these tests for a re-evaluation. The reason for this is that the fold structure used for the fold test [5] was a small scale and localized anticlinal structure. The test was conducted at the site level using sample data. Regionally the strata display minimal deformation with dips of less than 10°.

Lines 152-158, Table S1: apparently only the site KBA (sandstone) represents the clastic sediments in this study, others are carbonates. The KBA inclination is much less than inclinations in other sites (high-T component). Maybe there is something to do here with inclination compaction correction, which was found in some clastic rocks? Maybe if you exclude this site, the reversal test became positive?

Site KBA is indeed the only one from clastic sedimentary units, and its inclination is lower than those of the carbonate samples. It is possible that these inclinations are the product of inclination flattening due to compaction. However, exclusion of site KBA does not result in a positive reversal test, nor does it significantly change the high-temperature component mean. In the interest of transparency, we prefer to keep the result.

Lines 176-191 and Fig. S8(B): It would be much better to add a table (with ages) of used Indian poles in this supporting information.

We have added Table S2 listing Indian and North China poles (with their ages) used for comparison and reconstruction.

REVIEWERS' COMMENTS

Reviewer #1 (Remarks to the Author):

This is the second time that I have read this manuscript (once in its first submission, and now after revision). First, I want to thank the authors on their careful and considered revision and response to the initial reviewers. When reading a revision, I am always careful to read the response to reviewers to see that the authors have accommodated, or explained, all criticism.

In this case, I can clearly say that the primary criticisms have been adequately covered in the revision.

At this point, the only real drawback to the manuscript is driven by the short format of the work. The authors, however, have extensive supplementary information to round this out.

As before, I find this manuscript intriguing, and am pleased to now recommend it for publication.

Cheers,
Linda Kah

Reviewer #2 (Remarks to the Author):

The authors have done a good job of responding to my (minimal) comments on their original submission. But I am admittedly not an expert on these types of indirect indications of climate. I defer to the other reviewers, all of whom seem to have a better grip on the arguments than I do.

Reviewer #3 (Remarks to the Author):

Review of "High-latitude platform carbonate deposition…" (revised) by de Kock et al. submitted to Nature Communications

This revised version of the manuscript adequately addresses all of my comments except the one on paleomagnetism, which will be reviewer #4's call.
I am happy to note that the authors have toned down their extremism in using adjectives and adverbs, such as replacing "paradox" with "conundrum" and "very high latitude" with "high latitude".
Yes, I am comfortable with this version.

Abhijit Basu, Indiana University
January 17, 2024

Reviewer #4 (Remarks to the Author):

The corrections made by the authors are satisfactory. They properly addressed the issues I rose in my previous reviews. The corrections to remarks of other reviewers are also impro
